# Comparative and phylogenetic analyses of six Kenya *Polystachya* (Orchidaceae) species based on the complete chloroplast genome sequences

**DOI:** 10.1186/s12870-022-03529-5

**Published:** 2022-04-06

**Authors:** Hui Jiang, Jing Tian, Jiaxin Yang, Xiang Dong, Zhixiang Zhong, Geoffrey Mwachala, Caifei Zhang, Guangwan Hu, Qingfeng Wang

**Affiliations:** 1 CAS Key Laboratory of Plant Germplasm Enhancement and Specialty Agriculture, Wuhan Botanical Garden, Chinese Academy of Sciences, Wuhan, 430074 China; 2 Sino-Africa Joint Research Center, Chinese Academy of Sciences, Wuhan, 430074 China; 3 University of Chinese Academy of Sciences, Beijing, 100049 China; 4 East African Herbarium, National Museums of Kenya, P.O. Box 45166, Nairobi, 00100 Kenya

**Keywords:** *Polystachya*, Complete chloroplast genomes, Comparative genomics, Phylogenetic analysis

## Abstract

**Background:**

*Polystachya* Hook. is a large pantropical orchid genus (c. 240 species) distributed in Africa, southern Asia and the Americas, with the center of diversity in Africa. Previous studies on species of this genus have not obtained the complete chloroplast genomes, structures and variations. Additionally, the phylogenetic position of the genus in the Orchidaceae is still controversial and uncertain. Therefore, in this study, we sequenced the complete plastomes of six Kenya *Polystachya* species based on genome skimming, subjected them to comparative genomic analysis, and reconstructed the phylogenetic relationships with other Orchidaceae species.

**Results:**

The results exhibited that the chloroplast genomes had a typical quadripartite structure with conserved genome arrangement and moderate divergence. The plastomes of the six *Polystachya* species ranged from 145,484 bp to 149,274 bp in length and had an almost similar GC content of 36.9–37.0%. Gene annotation revealed 106–109 single-copy genes. In addition, 19 genes are duplicated in the inverted regions, and 16 genes each possessd one or more introns. Although no large structural variations were observed among the *Polystachya* plastomes, about 1 kb inversion was found in *Polystachya modesta* and all 11 *ndh* genes in the *Polystachya* plastomes were lost or pseudogenized. Comparative analysis of the overall sequence identity among six complete chloroplast genomes confirmed that for both coding and non-coding regions in *Polystachya*, SC regions exhibit higher sequence variation than IRs. Furthermore, there were various amplifications in the IR regions among the six *Polystachya* species. Most of the protein-coding genes of these species had a high degree of codon preference. We screened out SSRs and found seven relatively highly variable loci. Moreover, 13 genes were discovered with significant positive selection. Phylogenetic analysis showed that the six *Polystachya* species formed a monophyletic clade and were more closely related to the tribe Vandeae. Phylogenetic relationships of the family Orchidaceae inferred from the 85 chloroplast genome sequences were generally consistent with previous studies and robust.

**Conclusions:**

Our study is the initial report of the complete chloroplast genomes of the six *Polystachya* species, elucidates the structural characteristics of the chloroplast genome of *Polystachya*, and filters out highly variable sequences that can contribute to the development of DNA markers for use in the study of genetic variability and evolutionary studies in *Polystachya*. In addition, the phylogenetic results strongly support that the genus of *Polystachya* is a part of the tribe Vandeae.

**Supplementary Information:**

The online version contains supplementary material available at 10.1186/s12870-022-03529-5.

## Background

The genus *Polystachya* Hook (1824: 103; Orchidaceae) comprises approximately 240 species, of which most species are distributed in Africa, a few ones extend to tropical and subtropical Americas, and only three species in Asia [1–6]. It is difficult to give an exact number of species of the genus for while some species are widespread, others are narrow endemics. Furthermore, the taxonomic nomenclature of the genus is also complex since there are about 500 names available associated with the currently recognized species [2, 7]. Representatives of the genus are usually epiphytic, occasionally lithophytic or terrestrial perennial herbs [8, 9] (http://powo.science.kew.org/taxon/325981-2; http://www.theplantlist.org/tpl1.1/search?q=Polystachya). Most species of *Polystachya* are compact plants that take up little space with long-lasting and scented flowers, worth cultivating [8]. The ploidy levels of the genus range from diploids (2n = 2x = 40) to hexaploid (2n = 6x = 120), where it is generally considered to have evolved in the Neotropics, Madagascar and Reunion [10–12]. Previous studies indicated that there exists a close relationship between Polystachyinae and the tribe Vandeae. Based on morphological data analysis, Polystachyinae was placed within a larger Vandeae [13–15]. However, Carlsward et al. (2006b) maintained a stricter definition of Vandeae in their studies, citing several morphological characters of Vandeae, including monopodial habit, loss of mucilage and tilosomes, and the presence of spherical silica bodies in leaf sclerenchyma, to differentiate it from Polystachyinae [16]. A study by Li et al. (2019), using only one sample of *Polystachya* found that this genus belongs to Vandeae based on plastid genome sequence analysis. Nevertheless, the results of mitochondrial genome sequence analysis indicated that it may belong to Malaxideae, which is also supported by their shared morphological features such as distinctive pseudobulbs, terminal inflorescences, floral mentum and waxy pollinia [17].

The monophyly of the genus *Polystachya* has been reported in several studies, but it is uncertain whether its infrageneric taxonomic units are monophyletic [6, 18]. The genus was usually divided into 15 sections based on morphological characteristics, but these natural groupings are not fully supported in currently available molecular studies [6, 19, 20]. In these molecular studies, all the sections are either polyphyletic or paraphyletic, except for sect. *Isochiloides* (Russell et al., 2010b). A well-resolved phylogenetic hypothesis could help clarify the infrageneric classification of the genus and might be used to redefine sections as a step towards a much-needed generic revision. The latest taxonomic work to attempt an account of the entire genus was A Monograph of the Subtribe Polystachyinae Schltr. (Orchidaceae) edited by Mytnik-Ejsmont (2011) [18]. The species of the genus *Polystachya* are reclassified into 13 sections in the book. The classification system divided the sections of *Affines* (only *Polystachya affinis*), *Isochiloides*, *Dendrobianthe* (including *Polystachya dendrobiiflora*), and *Polystachya longiscarpa* (originally in *Dendrobianthe*) into separate genera. *Kermesina* and* Polystachate* were assigned to subsection levels. At present,* Polystachya dendrobiiflora* is an acceptable name, while *Dendrobianthe dendrobiiflora* is treated as its synonym, thus *Polystachya dendrobiiflora* is still used in this study. Apart from the sect. *Polystachya* which is pantropical, all sections are confined within Africa and Madagascar with two being endemic to Madagascar and the Indian Ocean islands. Although DNA sequence analysis has been informative in studies of the genus as a whole, relationships between members of this pantropical species group seem to play a lesser role. This is probably inseparable from the fact that the genus has a notoriously complicated taxonomy, with several sections that are widely used but probably not monophyletic [6, 12, 18, 19]. In addition, individuals from different geographical regions with duplicate samples may also not be monophyletic groups [21]. There has been some work on the genus, but their results rarely support the infrageneric taxonomic relationships currently delineated by Mytnik-Ejsmont, even with contradictions. Additionally, taxonomical and phylogenetic uncertainties remain in some sections or subsections or species because of poor internal resolutions, low bootstrap support, and incongruent plastid and nuclear gene phylogenies. It is generally believed that hybridization, polyploidization, ambiguous species definition, low sequence divergence level, reticulate evolution, incomplete lineage sorting, and plastid capture may complicate the issue for phylogenetic reconstruction of genus *Polystachya* [6, 12, 22]. Moreover, naming in *Polystachya* species is also complicated due to the influence of factors such as erroneous species identification, multiple synonyms, and highly variable morphological characters. Therefore, the development of more effective genetic resources on the basis of increasing samples is necessary for further phylogenetic studies of *Polystachya*. In recent years, an increasing number of researchers have focused on the cp genome to develop genetic markers for phylogenies. The cp genome sequences have been successfully used to evaluate relationships of different taxonomic units and yielded better results in handling the phylogenetic relations of many difficult groups [17, 23–31]. Accordingly, it is considered to be an informative and valuable resource for phylogenetic analysis in plants at multiple taxonomic levels.

Chloroplast (cp) is an important semi-autonomous organelle in plant cells with a complete genetic system and its genetic information is called chloroplast genome. The cp genomes in general are inherited uniparentally, and maternally in most angiosperms species at a slower evolutionary rate of change compared to nuclear genomes [32]. The typical plastomes in angiosperms have a highly conserved quadripartite circular structure comprised of a pair of inverted repeat regions (IRs, about 20–28 kb) and two single copy regions (large single copy region, LSC, 80–90 kb; small single copy region, SSC, 16–27 kb) [33]. These genomes range in sizes from 120 to 160 kb and usually encode 120–130 genes, with an overall GC content normally in the order of 30–40% [34–36]. Variation in genome size can be mainly attributed to IR expansion/contraction or even loss. The structure, length, GC content, gene type, gene content and order of cp genomes are generally conserved. When studying plant origin and phylogenetic relationships, the plant genome is made up of three parts: nuclear, mitochondrial and chloroplast genomes. Compared with nuclear and mitochondrial genomes, the chloroplast genomes are small, less prone to recombination, and have low rates of nucleotide substitutions, hence can provide distinct genetic information for phylogenetic studies [37, 38]. Furthermore, the phylogenetic reconstruction based on complete cp genome sequences may reduce errors and uncertainty resulting from insufficient sampling of DNA sequences [39].

With the rapid development of next-generation sequencing (NGS) technology, it is now more convenient and cheaper to obtain cp genome sequences, feasible to address various phylogenetic questions at the different taxonomic levels. Currently, over 6000 cp genomes of plants are available in the National Center for Biotechnology Information (NCBI) organelle genome database (https://www.ncbi.nlm.nih.gov), among which about 500 complete cp genome sequences (c. 370 species) of Orchidaceae have been released by NCBI (2021/5/20). Up to now, there is only one report on cp genome sequences of the genus *Polystachya* [21]. However, our reassembly of the original data from the report revealed that the cp genome sequences reported in that paper were both incomplete. Therefore, it is necessary to perform a comprehensive cp genomic comparison and phylogenetic analysis in the *Polystachya*.

In this study, we newly sequenced the complete chloroplast genomes of six *Polystachya* species and conducted comparative genomic analyses. Another 79 published cp genome sequences (77 from Orchidaceae; *Allium cepa* from Allioideae and *Iris gatesii* from Iridoideae were chosen as outgroups) downloaded from the NCBI database were used to construct phylogenetic trees. Our sampling Orchidaceae materials covered 5/5 subfamilies, 16/22 tribes and 20/49 subtribes of Orchidaceae. The objectives of this study are: (1) to report the first complete cp genome of the genus *Polystachya* and to reveal the structure and sequence variation of plastomes within *Polystachya*, (2) to reconstruct a more comprehensive and better-resolved phylogenetic tree for exploring the phylogenetic position of *Polystachya* in Orchidaceae and (3) to screen potential DNA markers in cp genomes that can be used for phylogenetic analysis and classification of *Polystachya*.

## Results

### Chloroplast genomes structure and features

**Fig. 1:**
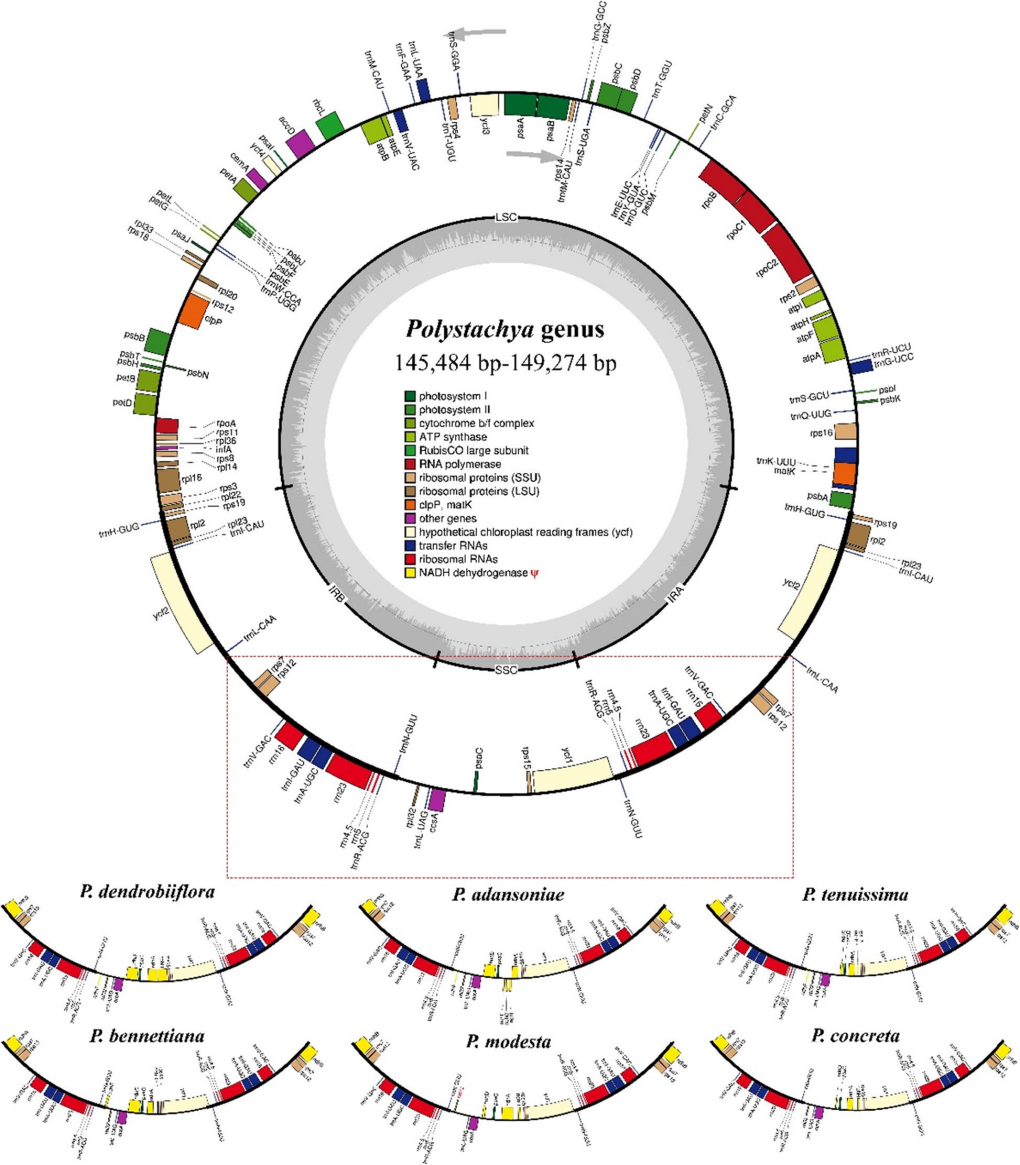
Chloroplast genome map of *Polystachya* species. The full circles represent common genes annotated for the six *Polystachya* species in this study, and the red empty box indicates differences in the genes annotated in this region of these plastomes. The bottom of the figure exhibits the differences in the genes annotated in this region for the plastomes. Genes shown outside the circle are transcribed in the counter counterclockwise direction, while those inside the circle are transcribed in the clockwise direction. The colored bars indicate genes belonging to different functional groups. The darker gray inside the inner circle denotes GC content while the lighter gray corresponds to the AT content of the genome. The ψ signifies pseudogenes

We obtained the six complete chloroplast genomes of *Polystachya* species, and these cp genomes ranged in sizes from 145,484 bp (*P. tenuissima*) to 149,274 bp (*P. dendrolliflora*). Like most angiosperms, all newly sequenced *Polystachya* plastomes displayed a typical quadripartite structure consisting of a pair of inverted repeats IR regions (IRA and IRB; 25,049–25,716 bp) separated by one large single copy region (LSC; 82,104–83,848 bp) and one small single copy region (SSC; 11,894–14,822 bp) **(**Fig. 1**,** Table 1**)**. The six cp genomes were all AT-rich, overall GC content ranged from 36.9 to 37.0%, and the GC content of IR region (43.2–43.3%) was always higher than that of LSC and SSC regions (34.3–34.5% and 28.8–29.5%, respectively) **(**Table 1**)**. This high GC percentage in the IR regions could be due to the presence of eight ribosomal RNA (rRNA) sequences in these regions. Previous studies have also shown similar results, with the high GC content in IR regions being related to the presence of all rRNA genes in this region [40]. By comparing all sequenced *Polystachya* cp genomes generated in this study, we found that they had highly conserved gene content, gene number, orientation and intron number.

**Table 1:** Chloroplast genome characteristics of six *Polystachya* species

Characteristics	*P. dendrobiiflora*	*P. adansoniae*	*P. tenuissima*	*P. bennettiana*	*P. modesta*	*P. concreta*
GenBank numbers	OK930071	OK930072	OK930073	OK930074	OK930075	OK930076
Voucher	HGW-M229	HGW-M230	HGW-M231	HGW-M232	HGW-M233	HGW-M234
Total length (bp)	149,274	147,680	145,484	145,995	148,853	146,717
LSC (bp)	83,848	83,450	82,104	82,669	83,475	82,718
SSC (bp)	14,822	14,132	12,192	11,894	14,622	13,179
IRs (bp)	25,302	25,049	25,594	25,716	25,378	25,410
Total number of genes	126 (107)	128 (109)	126 (107)	125 (106)	125 (106)	125 (106)
PCGs	74 (68)	74 (68)	74 (68)	74 (68)	74 (68)	74 (68)
tRNA	38 (30)	38 (30)	38 (30)	38 (30)	38 (30)	38 (30)
rRNA	8 (4)	8 (4)	8 (4)	8 (4)	8 (4)	8 (4)
Pseudogenes	6 (5)	8 (7)	6 (5)	5 (4)	5 (4)	5 (4)
GC content (%)	37.0	36.9	36.9	37.0	36.9	37.0
LSC (%)	34.4	34.3	34.3	34.3	34.4	34.5
SSC (%)	29.5	29.5	28.8	29.1	29.4	29.3
IR (%)	43.3	43.3	43.2	43.2	43.2	43.2

*LSC* large single copy region, *SSC* small single copy region, *IR* inverted repeat, *tRNA* transfer RNA, *rRNA* ribosomal RNA. *GC* guanine-cytosine. The numbers in parenthesis represent unique genes in the cp genomes.

A total of 106–109 unique genes were identified in the six cp genomes, including a shared 68 protein-coding genes (PCGs), 30 transfer RNA (tRNA) genes, and four ribosomal RNA (rRNA) genes **(**Table 1, Table S2). The difference in gene numbers were due to the variations in the number of ψ*ndh* among the six species. The gene distribution in these six cp genomes was exactly the same: the LSC regions encoded 59 protein-coding genes and 22 tRNA genes, and the SSC regions contained five protein-coding genes and one tRNA gene. Moreover, 19 genes were duplicated in IR regions, including six PCGs (*rpl2*, *rpl23*, *ycf2*, *rps19*, *rps7* and *rps12*), eight tRNA genes (*trnH*^-*CUG*^, *trnI*^-*CAU*^, *trnL*^-*CAA*^, *trnV*^-*GAC*^, *trnI*^-*GAU*^, *trnA*^-*UGC*^, *trnR*^-*ACG*^ and *trnN*^-*GUU*^), four rRNA genes (*rrn16*, *rrn23*, *rrn4.5* and *rrn5*) and one pseudogene (ψ*ndhB*) (Table S2). The remaining non-genic regions include introns, intergenic spacers (IGS), and pseudogene (ψ). Sixteen genes possessed introns: 13 genes (seven PCGs: *rps16*, *atpF*, *rpoC1*, *petB*, *petD*, *rpl16*, *rpl2*; six tRNAs: *trnK*^-*UUU*^, *trnG*^-*GCC*^, *trnL*^-*UAA*^, *trnV*^-*UAC*^, *trnI*^-*GAU*^ and *trnA*^-*UGC*^) have only one intron, while another three PCGs (*rps12*, *ycf3* and *clpP*) contain two introns (Table S2 and S3). Among the 16 intron-containing genes, 13 were present in LSC region and three were duplicated in the IR regions. At the same time, we found that the exon length was almost the same in the above 16 intron-containing genes, but the length of introns changed in all these genes. Interestingly, among the 11 plastid genes encoding the subunits of the NAD(P) H dehydrogenase complex (*ndh* genes), some genes were lost and some were pseudogenized (Fig. 1). The *trnK*^-*UUU*^ gene contains the longest intron, *matK* gene is located within *trnK*^-*UUU*^ intron. *Rps12* was a special trans-splicing gene, whose first exon is located in the LSC region, while the second and third exons reside in IR regions.

### Codon usage analyses

**Fig. 2:**
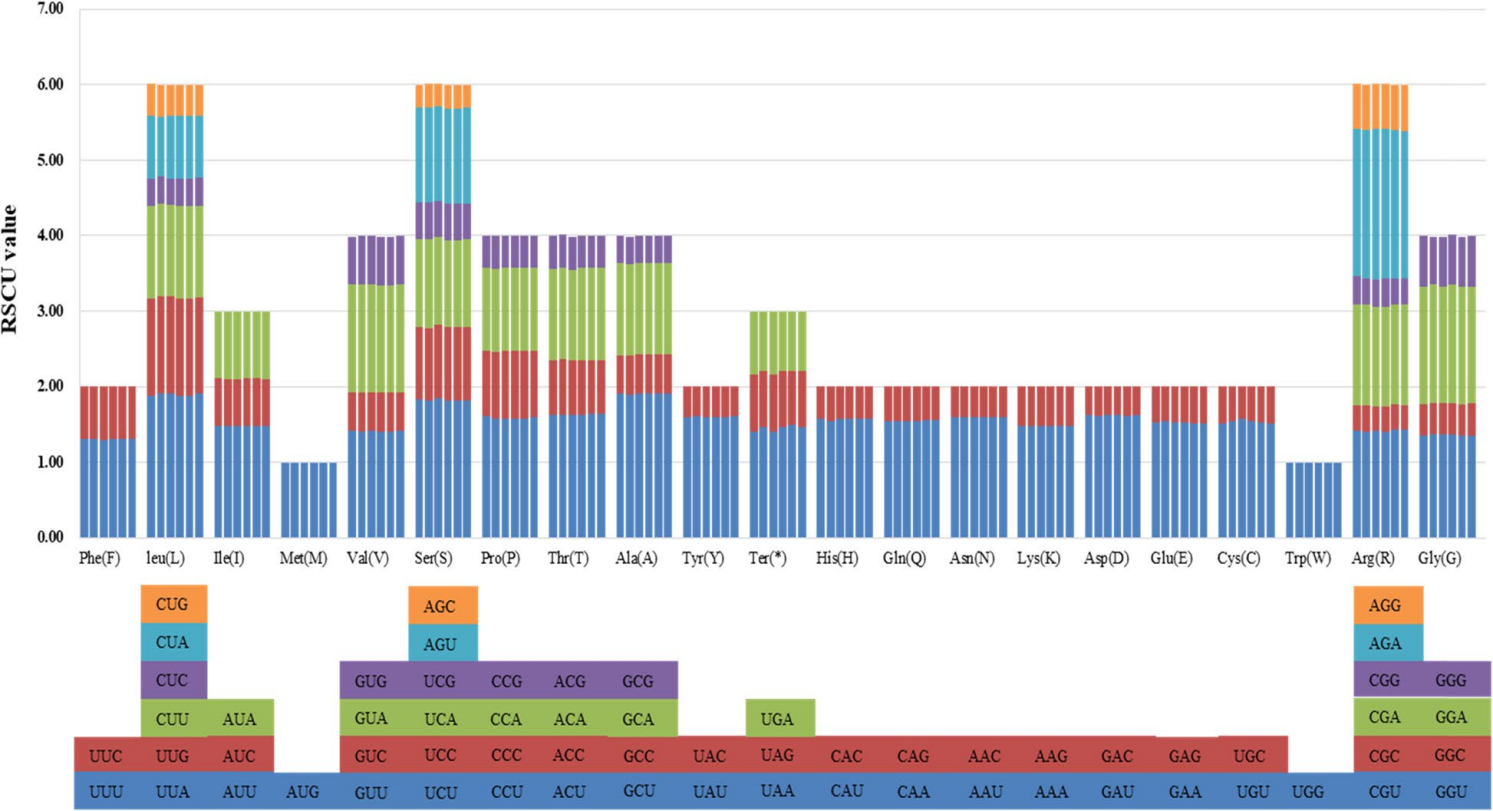
Codon content of 20 amino acids and stop codons in all protein-coding genes of the six cp genomes of *Polystachya* species. The histogram above each amino acid shows codon usage within *Polystachya*. Colors in the column graph reflect codons in the same colors shown below the figure. RSCU: relative synonymous codon usage; Ala: alanine; Arg: arginine; Asn: asparagine; Asp: asparagine; Cys: cysteine; Gln: glutamine; Glu: glutamic; Gly: glycine; His: histidine; Leu: leucine; Ile: isoleucine; Lys: lysine; Met: methionine; Phe: phenylalanine; Pro: proline; Ser: serine; Thr: threonine; Trp: tryptophan; Tyr: tyrosine; Val: valine

Codon usage frequency and relative synonymous codon usage (RSCU) were calculated based on protein-coding genes. All the 74 protein-coding genes were composed of 19,308–19,373 codons and encoded 20 amino acids in the chloroplast genomes of the six *Polystachya* species. **(**Fig. 2, Table S4). Among these amino acids, leucine (Leu; 1941–1955, 10.05–10.11%) is the most frequently used, cysteine (Cys; 209–216, 1.08–1.12%) is the least universal amino acid in the cp genomes of these species. The RSCU value analysis showed that almost all amino acids are encoded by 2–6 synonymous codons, except methionine (Met) and tryptophan (Trp), this strategy could protect protein mutations in biosynthesis. Relative synonymous codon usage is 1 for methionine (Met) and tryptophan (Trp). About half of the codons have RSCU > 1 (30/64), and most (29/30, 96.7%) end with the base A or T. Similarly, about half of the codons have RSCU < 1 (32/64), with the majority (29/32, 90.6%) ending with the base C or G. In nearly all of the protein-coding genes in *Polystachya* species had the standard ATG/CAT start codon (RSCU = 1), but *rpl2* started with ATA/TAT. ATA/TAT as an initiation codon has been reported in other cp genomes [41, 42]. All three stop codons were present, with TAA being the most frequently used among the six plastomes (Table S4).

### Repeat sequences analysis

**Fig. 3:**
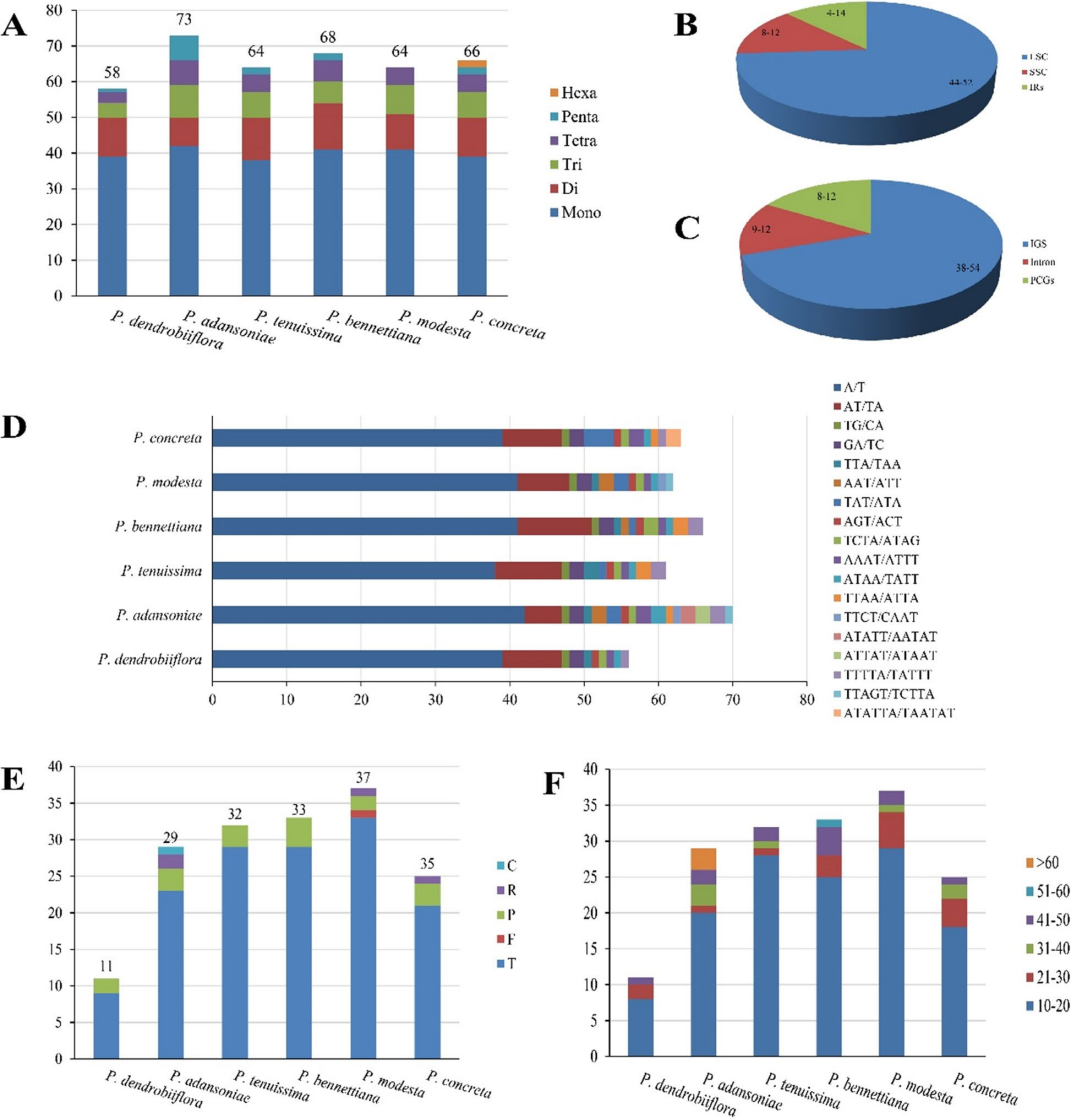
Analysis of repeated sequences of the six *Polystachya* cp genomes compared in this study. **A**: The number of six SSR types; (**B**): The number of SSRs distributed in different copy regions; (**C**): The number of SSRs distributed in different gene regions; (**D**): The number of different SSR repeat unit types; (**E**): The number of four long repeat types)

Simple sequence repeats (SSRs), also known as microsatellite repeats, are shorter tandem repeats consisting of 1–6 bp repeat units, which are widely distributed in various regions of chloroplast genome. In this study, a total of 58 (*P. dendrolliflora*)-73 (*P. adansoniae*) SSRs were detected in the six cp genomes, including 38–42 mononucleotides (mono-), 8–13 dinucleotides (di-), 4–9 trinucleotides (tri-), 3–7 tetranucleotides (tetra-), 0–7 pentanucleotide (penta-), and 0–2 hexanucleotide (hex) **(**Fig. 3A, Table S5). Among them, two hexanucleotides ATATTA/TAATAT distributed in IR regions only existed in *P. concreta* (Fig. 3D). Statistical analysis of the locations of all identified SSRs showed that the number of SSRs located in the LSC region, SSC region and IR regions were 44–52, 8–12 and 4–14, respectively. Furthermore, we found that these SSRs were mainly distributed in IGS (38–54), and some in the CDS region (9–12) and introns (8–12) (Fig. 3C). A/T (no C/G) is the only mononucleotide SSRs type in the six species, and the repeat units of the other five SSRs were also mainly composed of A or T **(**Fig. 3D). The tandem (T), forward (F), reverse (R), palindromic (P) and complement (C) repeat sequences in the six *Polystachya* cp genomes were conducted by tandem repeat finder and REPuter. We identified 11 (*P. dendrolliflora*)-37 (*P. modesta*) long repeat elements, of which 9–29 are tandem repeats (Fig. 3E, Table S6). The lengths of these long repeat sequences were variable with range 10–126 bp, with the longest repeat (126 bp) was presented in *P. adansoniae* (Fig. 3F, Table S6). Most tandem repeats were located in the IGS region of LSC, while the tandem repeats of the coding region were mainly located in the exons of *ycf1*, *ycf2*, *accD* and *rpoA* **(**Table S6).

### IR expansion and contraction

**Fig. 4:**
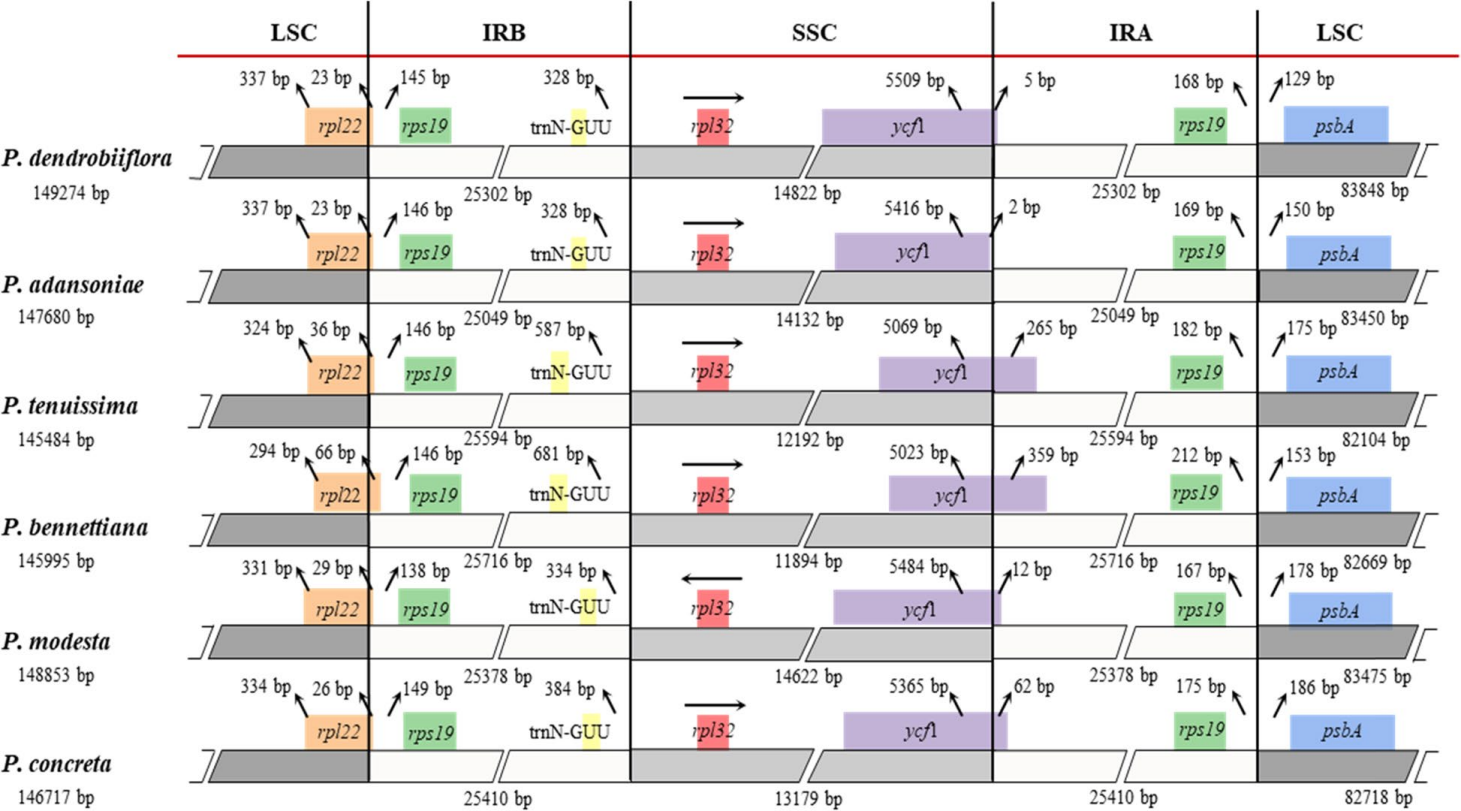
Comparisons of LSC, SSC and IR region borders among six *Polystachya* species cp genomes. Genes are denoted by colored boxes. The number above the gene features shows the distance between the end of the gene and the borders sites. The arrows indicate the location of the distance. This figure is not to scale

The contraction and expansion of IR borders are common evolutionary events and are the major reason for size differences between chloroplast genomes [43, 44]. We conducted a comparative analysis to investigate the expansion/contraction of IR among the six species, and found that the cp genomes of *Polystachya* species are greatly conserved. However, some structural variations were present in the four boundaries (LSC/IRB, IRB/SSC, SSC/IRA, IRA/LSC) **(**Fig. 4**)**. Our results showed that the genes *rpl22*-*rps19*-*psbA* and *trnN*-*rpl32*-*ycf1* were located in the junctions of the LSC/IR and SSC/IR regions. In all of the six plastomes, the *rpl22* gene spans the LSC/IRB junction region and extends to the IRA region for 23–66 bp.* Rps19* is duplicated in the IR regions, the distance between the *rps19* gene located in the IRB region and the *rpl22* gene is 138–149 bp, and the distance between the *rps19* gene located in the IRA region and the IRA/LSC boundary is 167–212 bp. IRB/SSC region is situated in the intergenic regions between *trnN*^-*GUU*^ and *rpl32*, the distance from *trnN*^-*GUU*^ to IRB/SSC region is 328–681 bp. In addition, the distance of *ycf1* gene varies greatly on the IRB/LSC border, with a length of 5–359 bp in the IR region and 5023–5509 bp in the SSC region. IRA/LSC region is situated in the intergenic regions between *rps19* and *psbA*, the distance from the *psbA* to the IRB /SSC boundary ranged from 129 to 186 bp.

### Structure comparison and divergence hotspot identification analysis

**Fig. 5:**
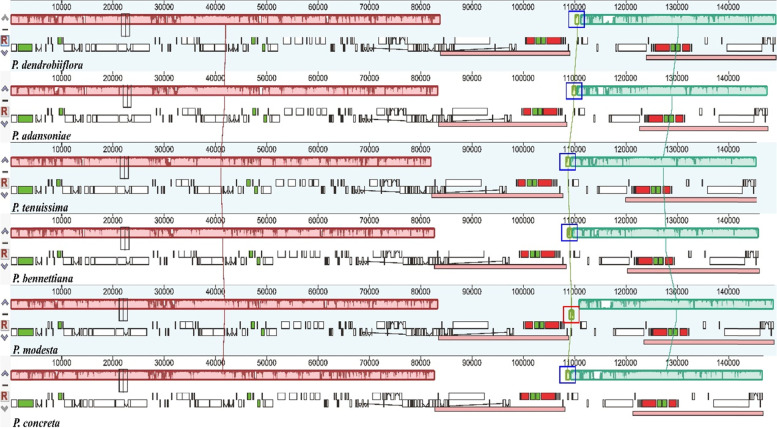
Intraspecific synteny analyses of six *Polystachya* species cp genome sequences. The chloroplast genome of *P. dendrobiiflora* is shown at the top as the reference sequence. Within each of the Mauve alignments, locally collinear blocks are represented by blocks of the same color connected by lines. In addition, the highlighted red box indicates that the sequence in this region has been reversed

**Fig. 6:**
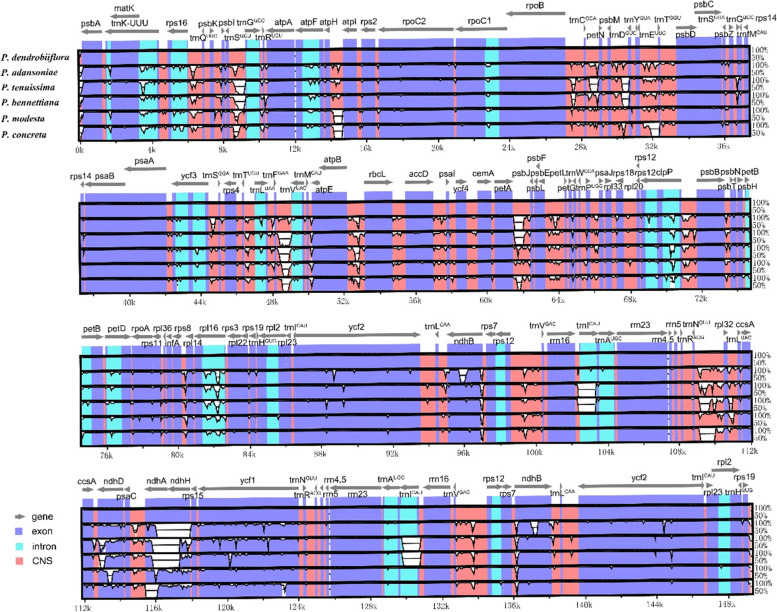
Sequence identity plot of six *Polystachya* species cp genome sequences. *P. dendrobiiflora* sequence as a reference, grey arrows indicate the orientation of genes, red bars represent non-coding sequences, purple bars represent exons, and blue bars represent introns; vertical scale indicates the percentage identity within 50–100%

**Fig. 7:**
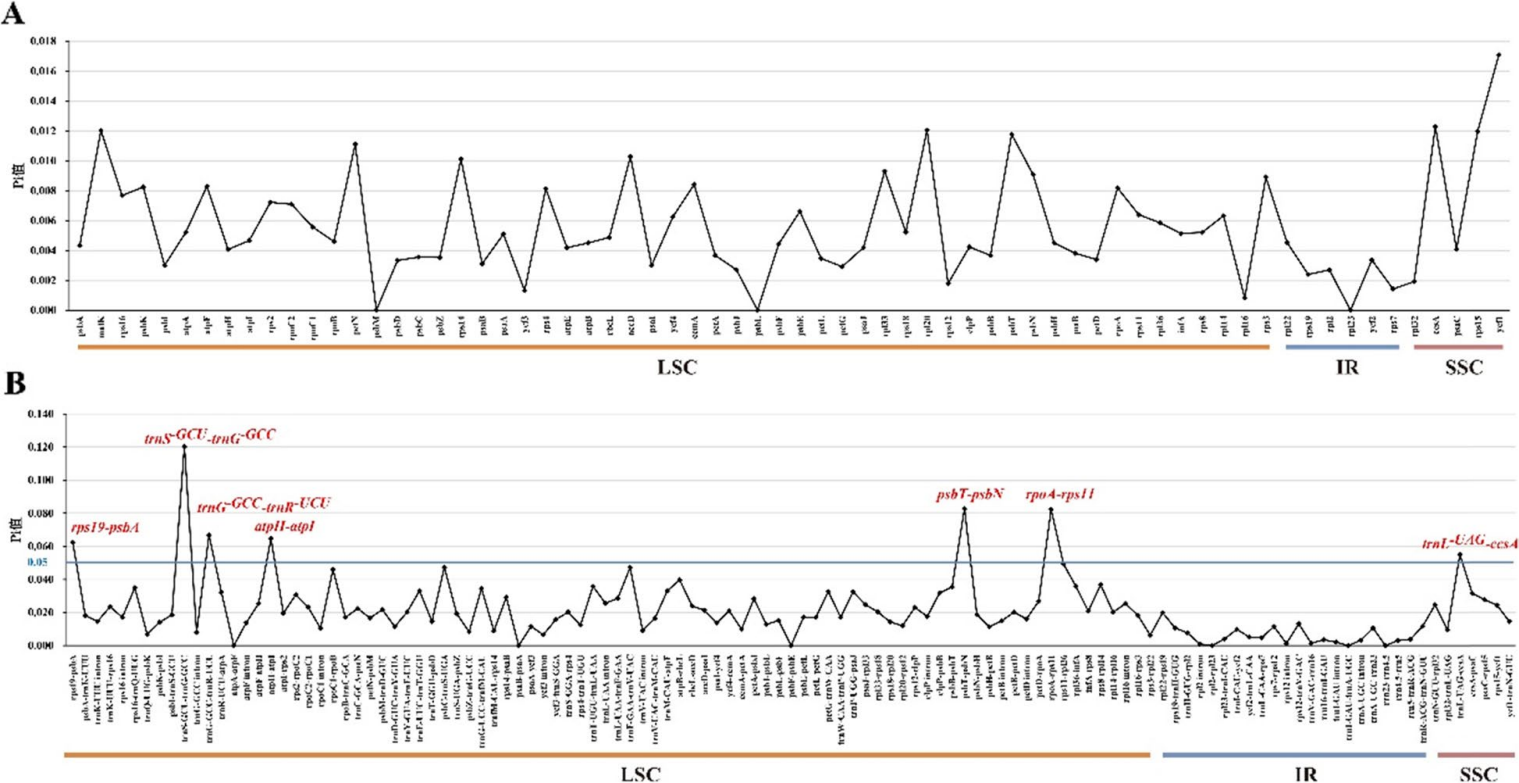
Sliding window analysis of six *Polystachya* cp genomes. (**A**) Comparison of the nucleotide variability (Pi) among protein-coding regions; (**B**) Comparison of the nucleotide variability among non-coding regions (including IGS and introns). X-axis: position of the midpoint of a window; Y-axis: nucleotide diversity of each window. The different colored lines at the bottom delineate where these genes are located in different plastome regions

Mauve comparison found a reversal of about 1 kb in the IRB/SSC boundary region of the cp genome of *P. modesta*, in which the *rpl32* gene was contained, resulting in the reversal of the *rpl32* gene **(**Fig. 5**)**. Sequence identity plots of the six *Polystachya* species were generated, with the annotation of *P. dendrobiiflora* chloroplast genome as a reference **(**Fig. 6**)**. LSC and SSC regions were more divergent than IRs regions. Whereas the coding regions were more conserved than the non-coding regions, the highly divergent non-coding regions among the six cp genomes appeared in the intergenic regions (IGS), such as *trnS*^-*GCU*^-*trnG*^-*GCC*^, *atpH*-*atpI*, *petA*-*psbJ*, *trnN*^-*GUU*^-*rpl32*, *rpl32*-*trnL*^*-*^^*UAG*^ and *psaC*-*rps15*. The *trnI*^-*GAU*^ intron was also relatively divergent. On the other hand, all the rRNA genes were highly conserved and were similar to other plants’ cp genomes [31]. For further understanding of the DNA polymorphism (Pi), the nucleotide variability value of 113 coding genes and 112 IGS regions were calculated among these cp genomes of the six *Polystachya* species **(**Fig. 7**)**. The results are the same as previous reports: the IR regions are more conserved than LSC and SSC regions and almost all divergent regions are presented in non-coding regions. The Pi values of the LSC and SSC regions were mostly greater than the largest Pi value (0.01149) of IR regions. There were seven variable regions with high Pi values (≥0.05), all which located in IGS regions, including *rps19*-*psbA* (0.06227), *trnS*^-*GCU*^-*trnG*^-*GCC*^ (0.12038), *trnG*^-*GCC*^-*trnR*^-*UCU*^ (0.06667), *atpH*-*atpI* (0.06479), *psbT*-*psbN* (0.08280), *rpoA*-*rps11* (0.08235) and *trnL*^-*UAG*^-*ccsA* (0.05503). These hotspot regions could be developed as molecular markers and used for DNA barcoding for future phylogenetic analyses and species identification of *Polystachya*. The Pi values received from coding regions ranged from 0.00000 to 0.01708 (*ycf1*) and the average value is 0.00556. However, IGS regions showed remarkably higher Pi values, the largest value was 0.12038 (*trnS*^-*GCU*^-*trnG*^-*GCC*^), with an average of 0.02158, which was 3.88-fold higher than that in coding genes.

### Positive selection analysis

The ratio of non-synonymous (dN) to synonymous substitutions (dS), dN/dS (ω), has been widely used to evaluate the natural selection pressure and evolution rates of nucleotides in genes [45, 46]. The ω ratio > 1 specifies positive selection (adaptive evolution), while ω ratio < 1 signifies negative selection (purifying evolution). We compared the ratio of non-synonymous (dN) and synonymous (dS) substitution for 68 shared protein-coding genes among six *Polystachya* species. Likelihood ratio tests (M7 vs. M8) supported the presence of positively selected codon sites (*p* < 0.05, *p* < 0.01) (Table S7). According to the M8 (beta & ω > 1) models, a total of thirteen genes are under significant positive selection in the Bayes Empirical Bayes (BEB) method, in which eight genes (*atpH*, *clpP*, *psbA*, *rbcL*, *rpl14*, *rpoA*, *rps3* and *ycf1*) harbored one significant positive selection site, three genes (*ccsA*, *matK* and *rpl16*) possessed two significant positive selection sites, while the *rpoC2* gene had five sites under positive selection **(**Table 2, Table S8). In addition, it was found that the *ycf2* gene located in the IR region had the highest number of positive selection sites, including 13 significant positive selection sites and one extremely significant positive selection site.

**Table 2:** Positive selection sites were detected in the cp genome of the six *Polystachya* species

M8							
Region	Gene Name	Selected Sites	Pr (ω > 1)	Region	Gene Name	Selected Sites	Pr (ω > 1)
LSC	*atpH*	1774G	0.955*	LSC	*rps3*	13885I	0.953*
SSC	*ccsA*	17025G	0.966*	SSC/IRA	*ycf1*	18,225 L	0.957*
		17026C	0.971*	IR	*ycf2*	15032C	0.952*
LSC	*clpP*	11,668 L	0.960*			15064A	0.951*
LSC	*matK*	441 T	0.959*			15339E	0.954*
		469Q	0.957*			15823S	0.957*
LSC	*psbA*	287R	0.961*			15839Q	0.956*
LSC	*rbcL*	9003I	0.958*			15,948 L	0.957*
LSC	*rpl14*	13618D	0.952*			16,192 L	0.951*
LSC	*rpl16*	13651F	0.950*			16216I	0.992**
		13739S	0.951*			16,224 K	0.954*
LSC	*rpoA*	13093Y	0.960*			16356R	0.958*
LSC	*rpoC2*	2429 L	0.953*			16395R	0.960*
		2512 M	0.957*			16402R	0.952*
		2529E	0.957*			16424Y	0.953*
		3003I	0.951*			16634I	0.950*
		3376Y	0.959*				

* *p* > 95%; ** *p* > 99%

### Phylogenetic analyses

**Fig. 8:**
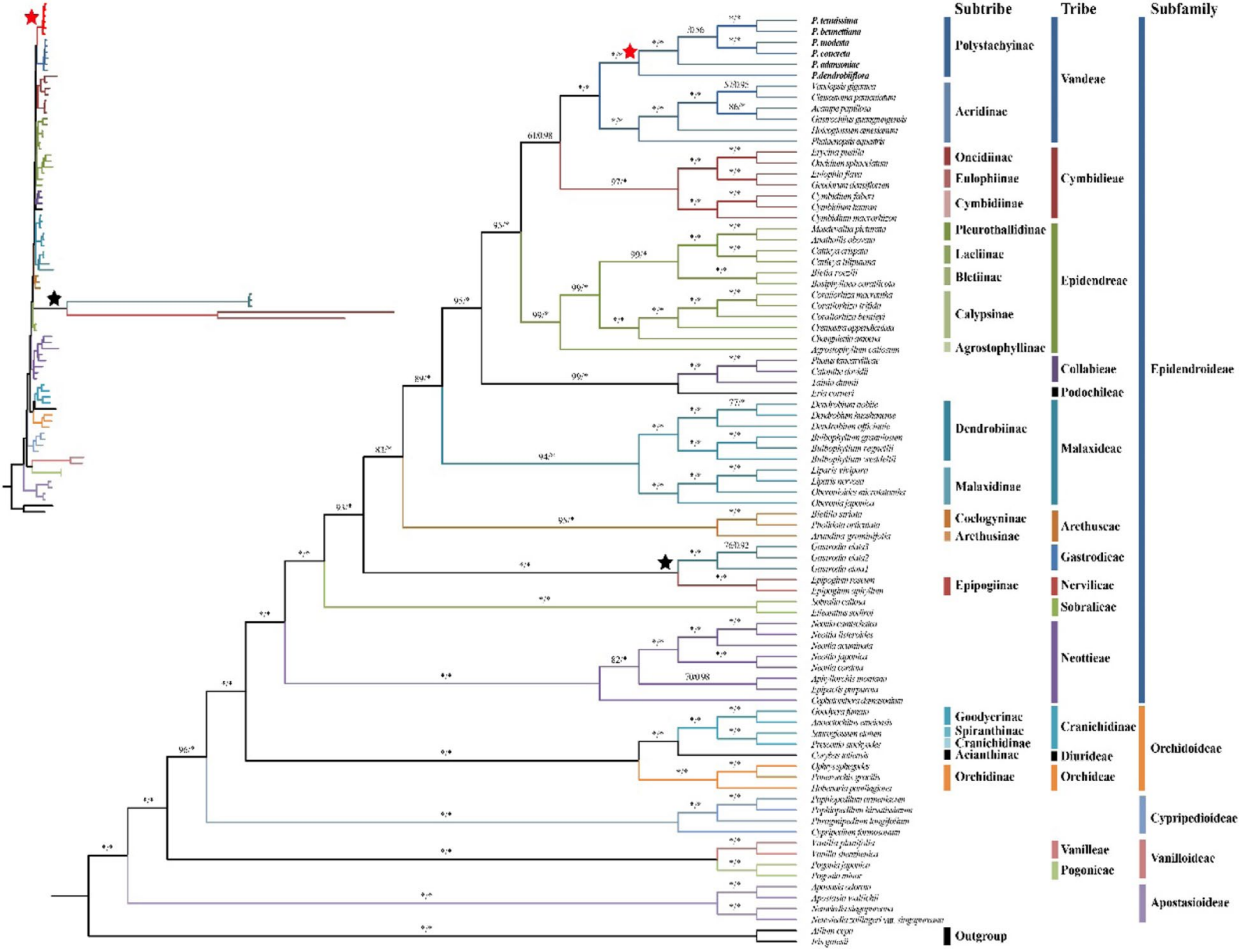
Phylogenetic tree constructed using Maximum Likelihood (ML) and Bayesian Inference (BI) methods, based on the 79 concatenate protein-coding sequences of whole cp genomes from 85 taxa. The numbers above the branches represent ML bootstrap values (BS)/BI posterior probabilities (PP). “*” indicates BS = 100% or PP = 1.00. “-” indicates BS < 50% or PP < 0.50. The figure in the upper left displays the branch lengths that indicate the distance relationships among the species

To investigate the phylogenetic position of *Polystachya* in the Orchidaceae family and the relationship among the *Polystachya* species, two datasets were extracted from 85 complete chloroplast genome sequences and used in this study. The phylogenetic trees generated from the two datasets were consistent except for the tribes of Vandeae, Cymbidieae and Epidendrea **(**Fig. 8, Table S10). In addition, the Maximum Likelihood (ML) and Bayesian Inference (BI) trees constructed from the same data set produce similar topologies. Therefore, phylogenetic trees constructed based on 79 CDS were selected for discussion in this study, and ML bootstrap (BS) and posterior probabilities (PP) values are given above branches **(**Fig. 8, Table S10). In all sampled species, *Polystachya* species formed a monophyletic clade with strong support (Fig. 8 and Table S10, BS, PP =100%, 1.00, respectively), where *P. dendrolliflora* is located at the base of this clade **(**Fig. 8, Table S10). Furthermore, the six *Polystachya* species and other all species of tribe Vandeae clustered together forming a monophyletic clade with strongly supported values (100%, 1.00). Based on the current data, our results yield a relatively stable phylogenetic relationship for most of the tribes and subtribes in Orchidaceae, with the exception of the tribes of Vandeae, Epidendrea and Cymbidieae. Additionally, the phylogenetic relationships of Orchidaceae at the subfamily level are fairly stable, with (Apostasioideae (Vanilloideae (Cypripedioideae (Orchidoideae, Epidendroideae)))) **(**Fig. 8, Table S10).

## Discussion

### Sequence variation

In this study, we collected six species of *Polystachya* and obtained their complete chloroplast genome sequences. In most angiosperm, the plastome is generally maternal inherited, with little recombination, and highly conserved structure [32, 47]. We found that the structure, gene content and gene order of the six *Polystachya* species were also highly conserved, and ranged in size from 145,484 bp (*P. tenuissima*) to 149,274 bp (*P. dendrolliflora*), containing 68 PCGs, four rRNA genes, and 30 tRNA genes **(**Table 1**,** Fig. 1**)**. The length of the LSC, SSC, and IRs varied in the range of 84,046–89,021 bp, 16,914–18,821 bp, and 23,902–25,914 bp, respectively. Chloroplast genome size variation among different species, or even within different individuals of the same species, which has been reported in other species, such as *Camptotheca acuminata* [48, 49], *Eucommia ulmoides* [50], *Rosa rugosa* [51–53] and *Calanthe davidii* [54]. This, besides polyploidy of plant material, has been shown to be due to the expansion/contraction of IR [55]. The GC content in six *Polystachya* species was almost similar (36.9–37.0%). Although the *Polystachya* cp genomes were AT-rich, the higher GC content in the IR region is most likely due to the presence of *rrn4.5*, *rrn5*, *rrn16*, and *rrn23* [56–58], which is consistent with the previously published Orchidaceae cp genomes [59, 60]. Non-coding regions, especially introns may have accumulated mutations more rapidly than coding regions, hence having an influence at gene expression level [61]. In this study, 16 genes possess introns among the six *Polystachya* species, and the lengths of their exons were almost the same, whereas the lengths of introns varied in all these genes. These changes in length may affect the size of the cp genome and gene expression. Moreover, *trnK*^-*UUU*^ gene containing the longest intron, and *rps12* are considered as a trans-splicing gene (Table S2), this identical phenomenon was consistent with those of most other members of Orchidaceae species [54, 62].

Inverted repeat (IR) expansion/contraction and sequence inversion represent important mechanisms of chloroplast genome rearrangement and show the diversity of cp genome structure in plants [63]. A detailed comparison of four IR/SC junctions of the six *Polystachya* plastomes showed that the border structures were highly similar to one another **(**Fig. 4**)**. Although the boundary regions of the cp genome sequences of the *Polystachya* species were relatively stable, we found expansion in IR regions, where *rpl22* gene in the LSC region was expanded by 23–66 bp to the IRB region. In addition, the *ycf1* gene in *P. adansoniae* was located exclusively in the SSC region with 2 bp away from the SSC/IRA border, whereas the *ycf1* gene in the SSC region expanded 5–359 bp into the IRA region in five other *Polystachya* species. The pseudogenization of *ycf1* gene is common in dicots since the IR/SC boundary is within the *ycf1* gene [26, 64], but only a short fragment of *ycf1* expanded into the IR region in this study, hence we did not annotate it as a pseudogene. An inversion of approximately 1 kb, within the IRB/SSC boundary region, was observed in the cp genome of *P. modesta*. However, the region corresponding to the inversion region was located in the SSC region in the remaining five species. This inversion caused the *rpl32* gene of *P. modesta* to reverse direction.

As a result of the high of mutation, the SSR markers were widely used for studies of genetic diversity, population structure and species authentication [65–67]. Moreover, repeat sequences were also considered as one of the important reasons affecting gene duplication, expansion, and genome rearrangement [68, 69]. A total of 58–73 SSRs and 11–37 long repeat sequences were identified, which were vastly distributed in the IGS region of LSC. The repeats in the coding regions were mainly located in the exons of *ycf1*, *ycf2*,* accD*, *ccsA*, *clpP*, *rpoA* and *rpoC2*. Most of the SSRs types are mononucleotide repeats, and A/T (no C/G) is the only mononucleotide SSRs type in the six *Polystachya* species. Previous research suggested that polyA and polyT have a more stable framework compared to polyC and polyG [70]. The large variation of long repeats in closely related species may reflect a certain degree of evolutionary flexibility [71]. The mVISTA percent identity plot and sliding window analysis showed that the most divergent regions were located in the *trnS*^-*GCU*^-*trnG*^-*GCC*^, *atpH*-*atpI*, *petA*-*psbJ*, *trnI*^-*GAU*^ intron, *trnN*^-*GUU*^-*rpl32*, *rpl32*-*trnL*^-*UAG*^ and p*saC*-*rps15* regions in the *Polystachya* plastomes. A comparative analysis of the six *Polystachya* species revealed that IR regions were mostly conserved and non-coding regions were more highly divergent than coding regions.

### NDH complex coding genes lost or pseudogenized

**Fig. 9:**
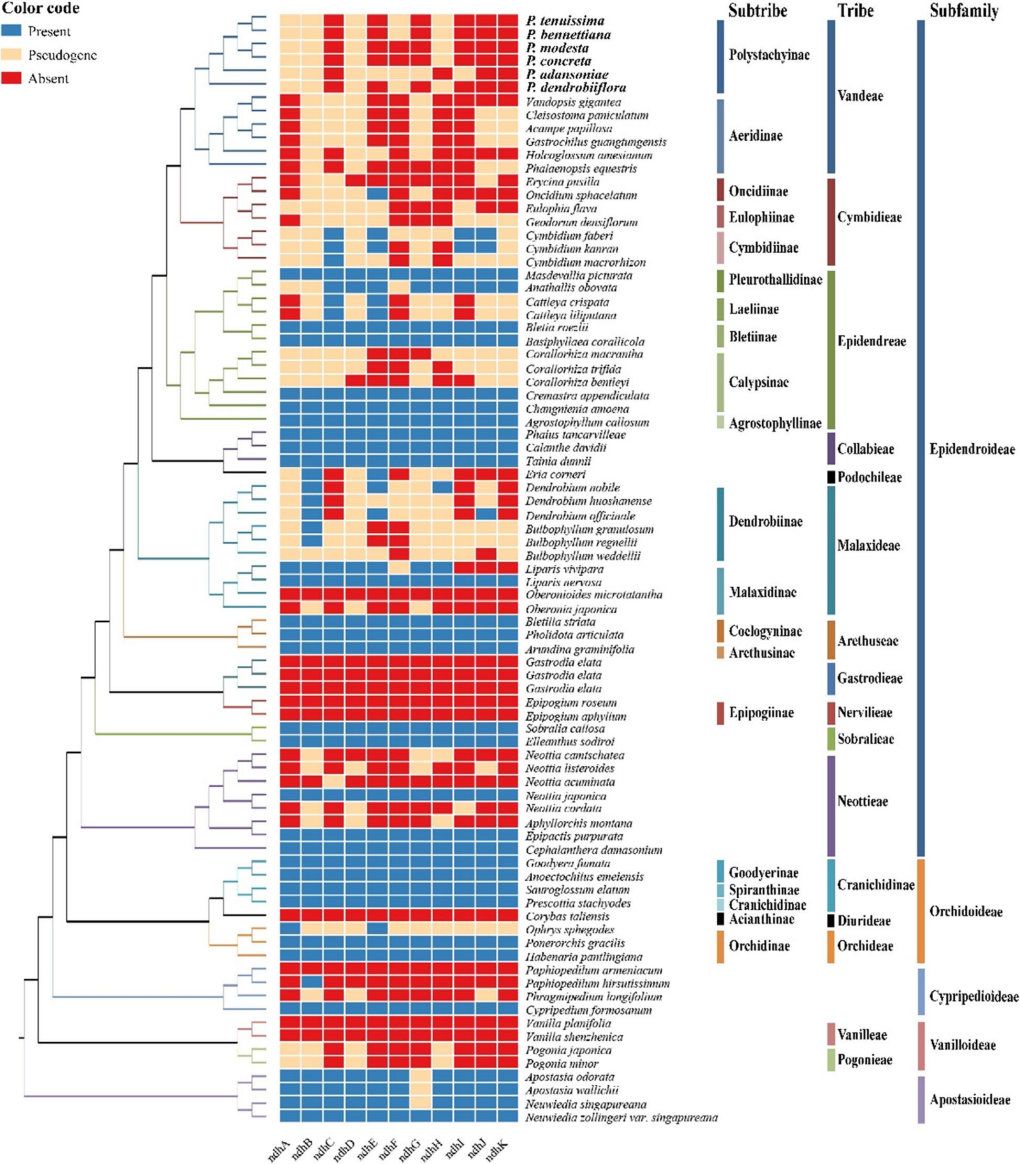
Distribution patterns of *ndh* gene loss in Orchidaceae. The blue, red, and yellow blocks indicate presence, pseudogene, and absence of each gene, respectively. 11 *ndh* non-normal genes are distributed widely across all taxonomic groups of Orchidaceae. Loss of *ndh* genes frequently occurs in Epidendroideae and Vanilloideae

The chloroplast NAD(P)H-dehydrogenase-like (NDH) complex is located in the thylakoid membrane and plays an important role in mediating photosystem I cyclic electron transport (PSI-CET) and facilitating chlororespiration. A chloroplast genome usually contains a total of 113 genes, comprising 6 *atp*, 11* ndh*, 6 *pet*, 9 *rpl*, 4 *rpo*, 12 *rps*, 4 *rrn*, 5 *psa*, 15 *psb*, 30 *trn* and 11 ungrouped genes. However, we found that 11 cp-*ndh* genes were lost or pseudogenized in the six *Polystachya* cp genomes **(**Fig. 9**)** and gradual pseudogenization, fragmentation and loss of *ndh* genes can be observed by comparative analyses of plastomes from the genus (Table S9). It is noteworthy that all *ndh* genes were also lost or pseudogenized in the plastomes of the sampled other species in the tribe Vandeae. Generally, *ndh* genes loss or pseudogenization is a common phenomenon in cp genomes of Orchidaceae, which is widespread in subfamilies Epidendroideae and Vanilloideae, and also occurs in subfamilies Cypripedioideae and Apostasioideae, but rare in the subfamily Orchidoideae. In addition, *ndh* genes are more frequently lost or pseudogenized in epiphytes than in terrestrial orchid plants [28, 72]. Considering, moreover, that most orchid species in which *ndh* gene non-functionalization has occurred retain photosynthetic capacity, it is inferred that the function of this gene class may be affected by the nuclear or mitochondrial genomes. It has been suggested that the gene function may be maintained by RNA editing after pseudogenization, but the possibility cannot be accurately assessed for the time being because most pseudogenizations involve not only base changes but also indels [28]. In general, *ndh* genes are more easily lost in saprophytic and lithophytic orchids than in epiphytic and terrestrial orchids. Nevertheless, there was no significant difference in the loss and pseudogenization of *ndh* genes between the saprophytic *Cymbidium macrorhizon* and the other two epiphytic orchids in the genus *Cymbidium*. Additionally, all *ndh* genes lost in the epiphytic *Oberonioides microtatantha*, *Corybas taliensis* and lost or pseudogenized in *Neottia cordata*. The *ndh* genes of the epiphytic *Agrostophyllum callosum* and *Pholidota articulata* were normal. However, we could not accurately determine whether the lifestyle of orchids is directly related to *ndh* gene evolution. Some studies have suggested that *ndh* gene loss in mycoheterotrophic orchid lineages (including leafy and photosynthetic orchids) may have not been so disadvantageous for the lineages that live in low-light canopy habitats as epiphytes, or in dark, understory habitats [73–75]. Furthermore, the loss and pseudogenization of the *ndh* gene is prevalent in heterotrophic plants [76] and is also widespread in other autotrophic plants such as conifers [77–80], Gnetales [80], Circaeasteraceae [81], *Corydalis* (Papaveraceae) [82], and *Erodium* (Geraniaceae) [83], which perhaps indicates that the loss of *ndh* genes might be a random phenomenon in photosynthetic lineage. The loss of plastome genes may be due to the transfer into the nucleus, substitution of a nuclear encoded mitochondrial targeted gene or substitution of a nuclear gene for a plastid gene [84–86]. It is not clear whether the *ndh* genes in *Polystachya* have been transferred to the nucleus or whether their loss represents the complete loss of the NDH complex.

### Divergent hotspots and adaptive evolution

The divergent regions as molecular markers could provide abundant valuable information for DNA barcoding and phylogenetic studies, and numerous phylogenetic reconstructions researches using divergent hotspots [66, 87]. We found that the nucleotide sequence diversity of the non-coding regions was higher than that of the coding regions, which is generally consistent with most previous studies on the chloroplast genomes of angiosperms [88]. In our study, we compared interspecific chloroplast diversity in six *Polystachya* species, which indicated that IR regions were mostly conserved and non-coding regions in the cp genome had most of the variation compared to protein-coding regions. This finding is generally consistent with most previous studies of the cp genomes of angiosperms [83]. Among the seven IGS regions (*rps19*-*psbA*, *trnS*^-*GCU*^-*trnG*^-*GCC*^, *trnG*^-*GCC*^-*trnR*^-*UCU*^, *atpH*-*atpI*, *psbT*-*psbN*, *rpoA*-*rps11* and *trnL*^-*UAG*^-*ccsA*) have high nucleotide diversity values (Pi > 0.05) which were most divergent regions identified **(**Fig. 7**)**. No significant mutations existed in IR regions. Based on the calculated Pi values for each gene region, we have conducted a comprehensive comparative analysis of all published chloroplast genomic data in *Polystachya*, and observed that IR region had a lower nucleotide diversity than LSC and SSC regions, *trnS*^-*GCU*^-*trnG*^-*GCC*^ with the highest variability values. These regions could potentially serve as universal candidate DNA barcodes for *Polystachya* species marker identification studies. It is noteworthy that the chosen divergent regions and the value of Pi are related to species selected, different collections were used, and different molecular markers will be selected out consequently. Thus, more suitable and accurate barcodes need to be further explored according to different sample categories.

The dN/dS (ω) ratios have been widely used to infer the evolutionary dynamics and identify adaptive signatures among species [89]. It has been indicated that the variation rates of the chloroplast genomes can be influenced under different environmental pressures, which may be the main reason for the differences in the number of genes in the cp genomes of some genera or species [90]. In our study, a total of 33 positively selected sites (including 32 significant and 1 extremely significant sites) were detected based on the BEB method, which were distributed in *atpH*, *ccsA*, *clpP*, *matK*, *psbA*, *rbcL*, *rpl14*, *rpl16*, *rpoA*, *rpoC2*, *rps3*, *ycf1* and *ycf2* genes **(**Table 2, Table S8). Of these loci, *ycf2* gene has the largest number of significant positively selected sites, and although it is the longest plastid gene reported in angiosperms and its function is yet largely unknown [91]. Despite this, *ycf2* provides a low-cost alternative to comprehensive multigene or genome datasets for investigating angiosperm relationships [92, 93]. Positive selection has also been found in *ycf2* in *Bulbophyllum* [94] and Malvaceae [95]. In addition, the genes *ccsA*, *clpP*, *matK*, *rbcL*, *rpl14*, *rpl16*, *rpoA*, *rpoC2*, *ycf1* and *ycf2* were identified in this study are also the targets of positive selection in many other flowering plants [95–97].

### Phylogenetic relationship

The chloroplast genomes have been widely employed as important data to resolve lineages within species phylogenetic analysis. Especially, the whole cp genomes or protein-coding regions have been considered more effective and accurate than the use of single gene sequences or several fragments [98, 99]. Consensus trees from BI and ML were almost similar, but the BI tree had higher resolution. *Polystachya* species formed a clade with strong support (BS, PP =100%, 1.00, respectively) based on the protein-coding sequences of cp genomes. The monophyly of the *Polystachya* has been similarly inferred in previous studies [6]. *P. tenuissima* (sect. *Cultriformes*) was sister to *P. bennettiana* (sect. *Caulescentes*), whereas *P. modesta* (sect. *Polystachya*) and *P. concreta* (sect. *Polystachya*) were sister taxa. These two clades are more closely related to each other than to* P. adansoniae* (sect. *Polychaete*). *P. dendrolliflora* represents the earliest extant lineage to diverge from the rest of the genus. Mytnik-Ejsmont (2011) proposed to divide *P. dendrolliflora* into an independent genus *Dendrobianthe*. Chase (2015) argued that there were no convincing arguments to split up the monophyletic and easily recognized genus *Polystachya* into smaller genera. Thus, *Dendrobianthe dendrobiiflora* is still treated as a synonym of *P. dendrobiiflora* (in TPL, POWO, IPNI). *P. modesta* is morphologically the most similar to pantropical tetraploid (including *P. concreta*) accessions and could be one of the parent species [12]. Morphological homology among species may be caused by introgression, plastid capture, hybridization and other factors, which makes it difficult for species delimitations in the genus *Polystachya*. Furthermore, the six *Polystachya* species and other all species of tribe Vandeae clustered together forming a monophyletic clade with strongly supported values (BS, PP = 100%, 1.00), which substantiated the definitive classification of this genus into Vandeae [13–15]. The phylogenetic relationships of all the sampled Orchidaceae species above subtribes are basically consistent with the results of Jin’s (2019) study based on cpCDS, except that the phylogenetic positions among the tribes of Vandeae, Epidendrea and Cymbidieae are slightly different [17]. Although all phylogenetic relationships cannot be resolved by using only the complete cp genomes, our results suggest that plastome-wide analysis will provide higher a resolution for some disputed taxonomic relationships. In addition, the genus has a notorious taxonomic problem that has not been well resolved below the genus level [6, 12, 18]. Previous phylogenetic studies of this genus were mainly based on a few plastids and nuclear gene fragments, and although these results were able to roughly locate most taxonomic units, the phylogenetic relationships constructed by selecting different fragments were biased and many branches had low support. The chloroplast genome sequences with higher resolution provided by our study may provide a promising perspective for further elucidating the phylogeny and evolution within the *Polystachya* genus.

## Conclusions

In this study, we obtained the complete chloroplast genomes of six *Polystachya *species (*Polystachya adansoniae*, *P. bennettiana*, *P. dendrobiiflora*, *P. tenuissima*, *P. modesta* and *P. concreta*) and revealed that the overall structure and gene content of the plastomes of the six species are relatively conserved, with only some variations in genome sizes, gene content, GC contents, introns, repeats and IR borders. It is interesting to note that all *ndh* genes were lost or pseudogenized in the plastomes of *Polystachya*, and were also observed in sampled other species in the tribe Vandeae. We also identified 13 positive selection genes and 16 variable regions, which provide a reference for developing DNA markers and adaptive evolution to further studies of *Polystachya* species. Phylogenetic analysis based on the current data identified the genus *Polystachya* in the tribe Vandeae and largely established the phylogenetic relationships of most taxonomic units in tribe and above tribe of Orchidaceae. These findings not only strengthen our understanding of the *Polystachya* plastomes, but also contribute to our further appreciation of the phylogenetic position of *Polystachya* in the family Orchidaceae.

## Materials and methods

### Samplings and DNA extraction

**Fig. 10:**
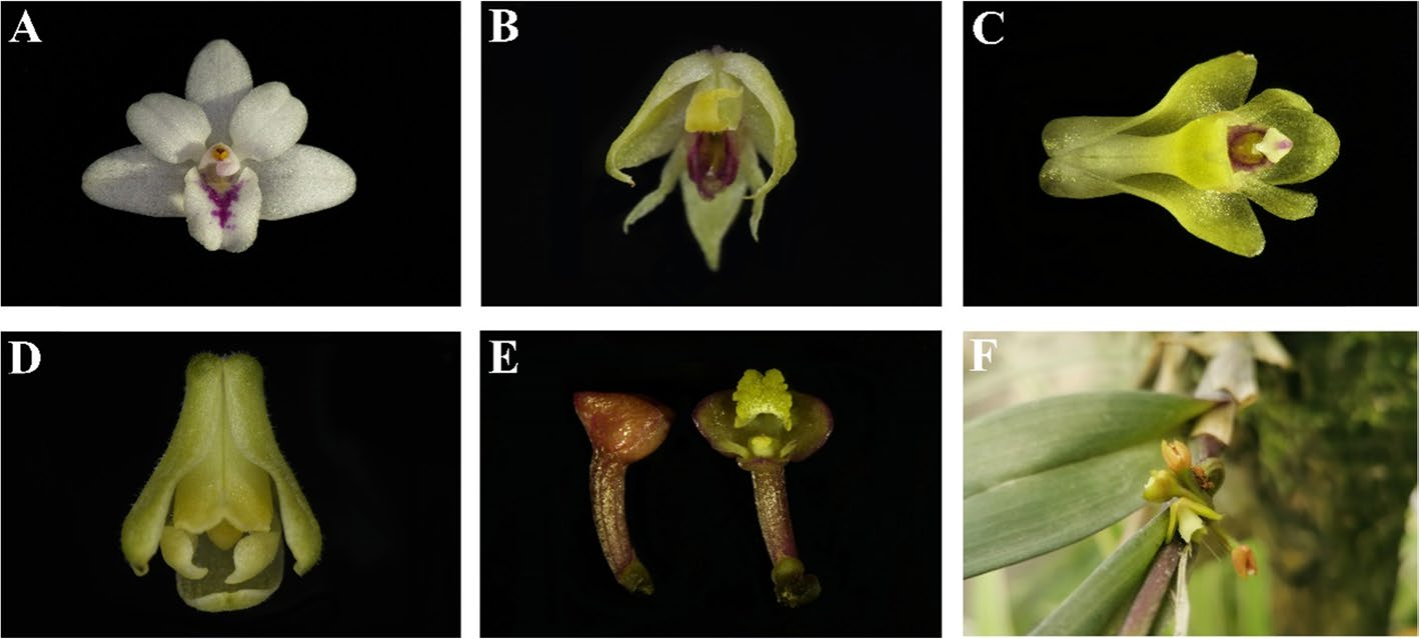
Photographs of six* Polystachya* species. **A**. *Polystachya dendrobiiflora* Rchb.f. (Kenya, Coast Province); (**B**). *P. adansoniae* Rchb.f (Kenya, Rift Valley Province); (**C**). *P. tenuissima* Kraenzl. (Kenya, Cherangani Hills West Pokot County); (**D**). *P. bennettiana* Rchb.f. (Kenya, Rift Valley Province); (**E**). *P. modesta* Rchb.f. (Kenya, Coast Province); (**F**). *P. concreta* (Jacq.) Garay & H.R.Sweet (Kenya, Coast Province). Photos by Guangwan Hu and Hui Jiang

Plant materials of the six *Polystachya* species: *Polystachya dendrobiiflora* Rchb.f. (voucher number: HGW-M229), *Polystachya adansoniae* Rchb.f. (voucher number: HGW-M230), *Polystachya tenuissima* Kraenzl. (voucher number: HGW-M231), *Polystachya bennettiana* Rchb.f. (voucher number: HGW-M232), *Polystachya modesta* Rchb.f. (voucher number: HGW-M233) and *Polystachya concreta* (Jacq.) Garay & H.R.Sweet (voucher number: HGW-M234) were collected from Kenya in the joint field investigations performed by the National Museums of Kenya (NMK) and Sino-Africa Joint Research Center, CAS (SAJOREC) during 2015 to 2018 **(**Fig. 10**)**. Young and fresh leaves were sampled and immediately preserved using silica gel [100]. The voucher specimens were deposited in the East African Herbarium (EA) at the National Museums of Kenya and the Herbarium of Wuhan Botanical Garden, CAS (HIB). Total genomic DNA was extracted using a modified cetyltrimethylammonium bromide (CTAB) method [101]. DNA integrity was examined by electrophoresis in 1% (w/v) agarose gel, their purity was determined using a NanoDrop spectrophotometer 2000 (Thermo Scientific; Waltham, MA, USA) at 260 and 280 nm, and precisely quantify DNA concentration with Qubit 2.0 (Life Technologies, CA, USA).

### High throughput sequencing, genome assembly and annotation

The purified high-quality genomic DNA was used to construct paired-end (PE) libraries by shearing the genomic DNA into short fragments of approximately 350 bp before sequencing in 150 bp paired-end mode was implemented on an Illumina HiSeq 2500 platform (Illumina, Inc., San Diego, CA, USA) at Novogene Company (Tianjin, China). Genomes assembly were performed using GetOrganelle v1.7.1 [102] and CLC Genomic Workbench v10 (CLC Bio., Aarhus, Denmark) with the default parameters. The quality of the newly assembled genomes was evaluated on read level basis by aligning the trimmed raw reads to the de novo assemblies using Geneious mapper, Geneious v8.0.2 with medium- to low-sensitivity option and iteration up to five times [103, 104]. The resulting complete chloroplast genomes were automatically annotated using the Perl script Plastid Genome Annotator (PGA) and the Annotation of Organellar Genomes (GeSeq) (https://chlorobox.mpimp-golm.mpg.de/geseq.html) [105, 106]. Further annotation confirmation was performed with published genomes the cp genomes of the tribe Vandeae, Cymbidieae and Epidendreae sampled species in this study were used as the reference sequences. According to the annotation results of the two softwares, manual corrections of start/stop codons and intron/exon boundaries were performed in Geneious v8.0.2. All transfer RNA (tRNA) genes were proofread with the web server tRNAscan-SE v2.0 (http://lowelab.ucsc.edu/tRNAscan-SE/) [107]. A gene was classified as a pseudogene if its reading frame was truncated (incl. Due to a premature stop codon) or frameshifted compared with Orchidaceae species [108]. Physical maps of the circular plastomes were visualized using the Organelle Genome DRAW (OGDRAW) (http://ogdraw.mpimp-golm.mpg.de/) [109]. All annotated complete cp genome sequences were deposited into GenBank under the accession numbers listed in Table 1 and Table S1.

### Repeat sequences analysis

Simple sequence repeat (SSR) markers were identified in these plastome sequences using Phobos v3.3.12 [110] and SSR Hunter v1.3 [111] with minimum repeat thresholds of 10 for mononucleotide (mono-) repeats, 5 for dinucleotide (di-) repeats, 4 for trinucleotide (tri-) repeats, and 3 for tetranucleotide (tetra-), pentanucleotide (penta-), and hexanucleotide (hexa-) repeats. The size and location of larger repeat sequences including forward (F), palindromic (P), and reverse (R) repeats were searched using the online program REPuter (https://bibiserv.cebitec.uni-bielefeld.de/reputer) [112] with the set as: Hamming distance of 3 and minimum repeat size of 30 bp. Tandem Repeats Finder v4.07 (https://tandem.bu.edu/trf/trf.html) [113] was employed to discover tandem repeats (T) using default settings.

### Comparison and divergence hotspot identification analysis

To investigate the structure difference exists, the expansion/contraction of the IR regions was assessed by comparing the positions of SC/IR junctions and their adjacent genes using IRscope [114], complete chloroplast genomes were aligned with MAFFT v7 [115]. The online mVISTA program (http://genome.lbl.gov/vista/mvista/submit.shtml) [116] was employed to compare the whole cp genome divergence within six *Polystachya* species in Shuffle-LAGAN mode, and with *Polystachya dendrobiiflora* as the reference. Genome rearrangement, inversions and gene synteny was detected using MAUVE [117] with default settings. All protein-coding genes of each genome sequence were extracted in Geneious v8.0.2 to examine the relative synonymous codon usage (RSCU) using MEGA v7.0 [118]. The codon usage frequency and relative synonymous codon usage (RSCU) of the six species were conducted based on 68 PCGs using MEGA v7.0. The nucleotide diversity (Pi) of each plastome was implemented to evaluate by DnaSP v6 [119].

### Positive selection analysis

To detect the protein-coding genes under selection in the six *Polystachya* species, we calculate the non-synonymous (dN) and synonymous (dS) substitution rates utilizing the CodeML algorithm implemented in EasyCodeML [120]. Each single-copy CDS was aligned separately using codon mode under the MAFFT v7 [115], followed by checking each single gene matrix and manually adjust abnormal codon alignments. Subsequently, these alignments were concatenated into a matrix using PhyloSuite v1.1.15 [121]. The ML tree was constructed using IQ-TREE [122] as an input tree. In preset mode, we used site model to test for natural selection, and eight codon substitution models described as M0 (one-ratio), M1a (nearly neutral), M2a (positive selection), M3 (discrete), M7 (beta), M8 (beta & ω > 1) and M8a (beta & ω = 1) were investigated. This model allowed the ω ratio to vary among sites with a fixed ω ratio in all branches in order to test for site-specific evolution in the gene phylogeny. The likelihood ratio test (LRT) was performed to detect positively selected sites with four pairs of site-specific models: M0 vs M3, M1a vs. M2a, M7 vs. M8, and M8a vs. M8. Comparing the four pairs of site-specific models, M7 vs. M8 was calculated to identify positive selection sites based on both ω and LRTs values. The Bayes empirical Bayes (BEB) method was used to statistically identify sites under positive selection with posterior probabilities ≥0.95 [123].

### Phylogenetic analysis

To ascertain the phylogenetic position of *Polystachya* within Orchidaceae, a total of 85 species were analyzed, of which six *Polystachya* species were newly sequenced in this study and other 77 cp genome sequences of Orchidaceae species downloaded from NCBI database. Moreover, these sampled Orchidaceae materials represented 5/5 subfamilies, 16/22 tribes and 20/49 subtribes of Orchidaceae. According to previous studies [17, 28], *Allium cepa* (MK335926, Allioideae) and *Iris gatesii* (KM014691, Iridoideae) were chosen as outgroups. GenBank accession numbers and detailed information of all samples used in this study are listed in Table S1. Each protein-coding sequence (CDS) matrix alignment was performed using MAFFT v7 plugin integrated into PhyloSuite v1.1.15 [121]. An incongruence length difference (ILD) test was performed in PAUP v4.0a168 [124] to determine whether data from different genes can be combined, and this test indicated that these datas are not homogeneous (*P* < 0.01). All alignments were eventually concatenated into one supermatrix utilizing PhyloSuite v1.1.15 [121]. Substitutional saturation was assessed using DAMBE v 7.0.68 [125] for the concatenated matrix. Subsequently, the 79 CDSs and the first and second codon positions of 79 CDSs were used, and the phylogenetic trees were constructed by Maximum Likelihood (ML) and Bayesian Inference (BI) algorithms. ML phylogenies were conducted using RAxML v8.2.12 [126] with 1000 bootstrap replicates and the GTRGAMMA model. BI phylogenies were inferred using MrBayes 3.2.6 [127] with the best-fitting substitution model by ModelFinder [128]. The Markov Chain Monte Carlo (MCMC) algorithm was run for 2,000,000 generations, sampling every 1000 generations, in which the initial 25% of sampled data are discarded as burn-in. Samples were combined and convergence of chains was checked in Tracer v1.7.2 [129]. Figtree v1.4.4 (http://tree.bio.ed.ac.uk/software/figtree/) was used to visualize and annotate trees.

## Supplementary Information

**Additional file 1: Table S1.** Taxonomic and GenBank accession information forsamples used for phylogenetic analyses (85). **Table S2 and S3.** Types of genes annotation and the intron-containing genes within the chloroplastgenomes of six *Polystachya* species. **Table S4.** Codonusage within the chloroplast genomes of six *Polystachya*species. **Table S5.** Typesand amounts of SSRs within the chloroplast genomes of six *Polystachya *species. **Table S6.** Locationof repeat sequences within the chloroplast genomes of six *Polystachya* species. **Table S7.** Comparisonof site models for the 68 shared CDSs in the chloroplast genomes of six *Polystachya*species and results of LRT. **Table S8.** Positiveselection sites based on BEB analysis in the M8 model detected in thechloroplast genomes of six *Polystachya* species. **Table S9.** Theoverall view of all gene alignment in the complete chloroplast genomes of six *Polystachya*species. **Table S10.** Phylogenetictree constructed using ML and BI methods, based on the first and second codonpositions of 79 CDSs of whole cp genomes from 85 taxa.

## Data Availability

All the newly sequenced sequences in this study are available from the National Center for Biotechnology Information (NCBI) (accession numbers: OK930071-OK930076; see Table 1 and Additional Table 1: Table S1). Information for other samples used for phylogenetic analysis download from GenBank can be found in Additional Table 1: Table S1.

## Abbreviations

NGS: Next-generation sequencing
cp: Chloroplast
LSC: Large single copy
SSC: Small single copy
SC: Single copy
IR: Inverted repeat
CDS: Coding sequence
RSCU: Relative synonymous codon usage
SSR: Simple sequence repeats
Pi: DNA polymorphism
ML: Maximum likelihood
BI: Bayesian Inference
