# Supplementary material for: Comparative and phylogenetic analyses of six Kenya Polystachya (Orchidaceae) species based on the complete chloroplast genome sequences
Source: BMC Plant Biol. 2022 Apr 6;22:177. doi: 10.1186/s12870-022-03529-5 (PMC8985347; doi:10.1186/s12870-022-03529-5)
Supplement: Supplementary file 1 — Additional file 1: Table S1. Taxonomic and GenBank accession information forsamples used for phylogenetic analyses (85). Table S2 and S3. Types of genes annotation and the intron-containing genes within the chloroplastgenomes of six Polystachya species. Table S4. Codonusage within the chloroplast genomes of six Polystachyaspecies. Table S5. Typesand amounts of SSRs within the chloroplast genomes of six Polystachya species. Table S6. Locationof repeat sequences within the chloroplast genomes of six Polystachya species. Table S7. Comparisonof site models for the 68 shared CDSs in the chloroplast genomes of six Polystachyaspecies and results of LRT. Table S8. Positiveselection sites based on BEB analysis in the M8 model detected in thechloroplast genomes of six Polystachya species. Table S9. Theoverall view of all gene alignment in the complete chloroplast genomes of six Polystachyaspecies. Table S10. Phylogenetictree constructed using ML and BI methods, based on the first and second codonpositions of 79 CDSs of whole cp genomes from 85 taxa. [file 12870_2022_3529_MOESM1_ESM.zip › Table S10.docx]

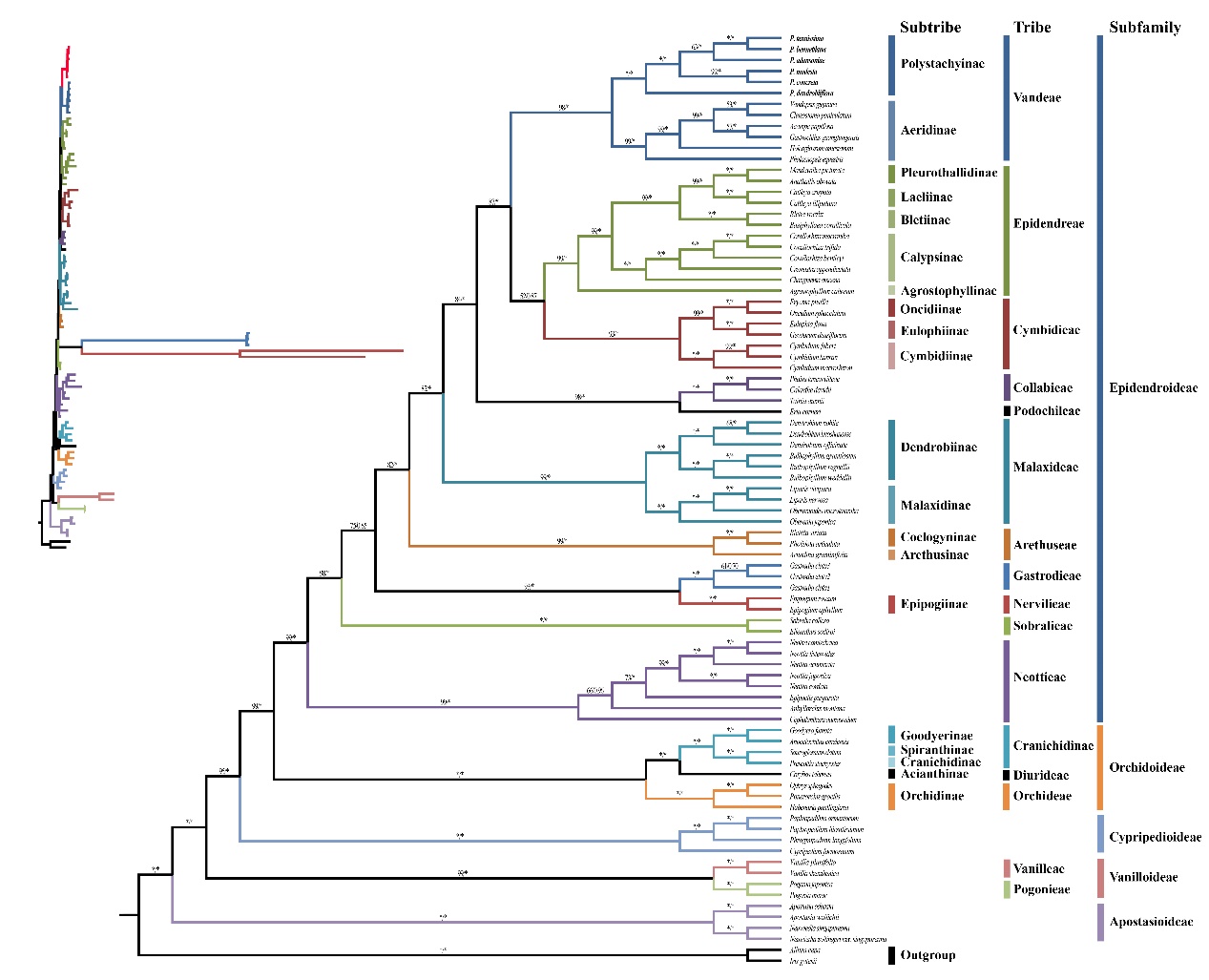


Figure S1. Phylogenetic tree constructed using ML and BI methods, based on the first and second codon positions of 79 CDSs of whole cp genomes from 85 taxa. The numbers above the branches represent ML bootstrap values (BS)/BI posterior probabilities (PP). “*” indicates BS=100% or PP=1.00. “-” indicates BS<50% or PP<0.50. The figure in the upper left displays the branch lengths that indicate the distance relationships among the species.


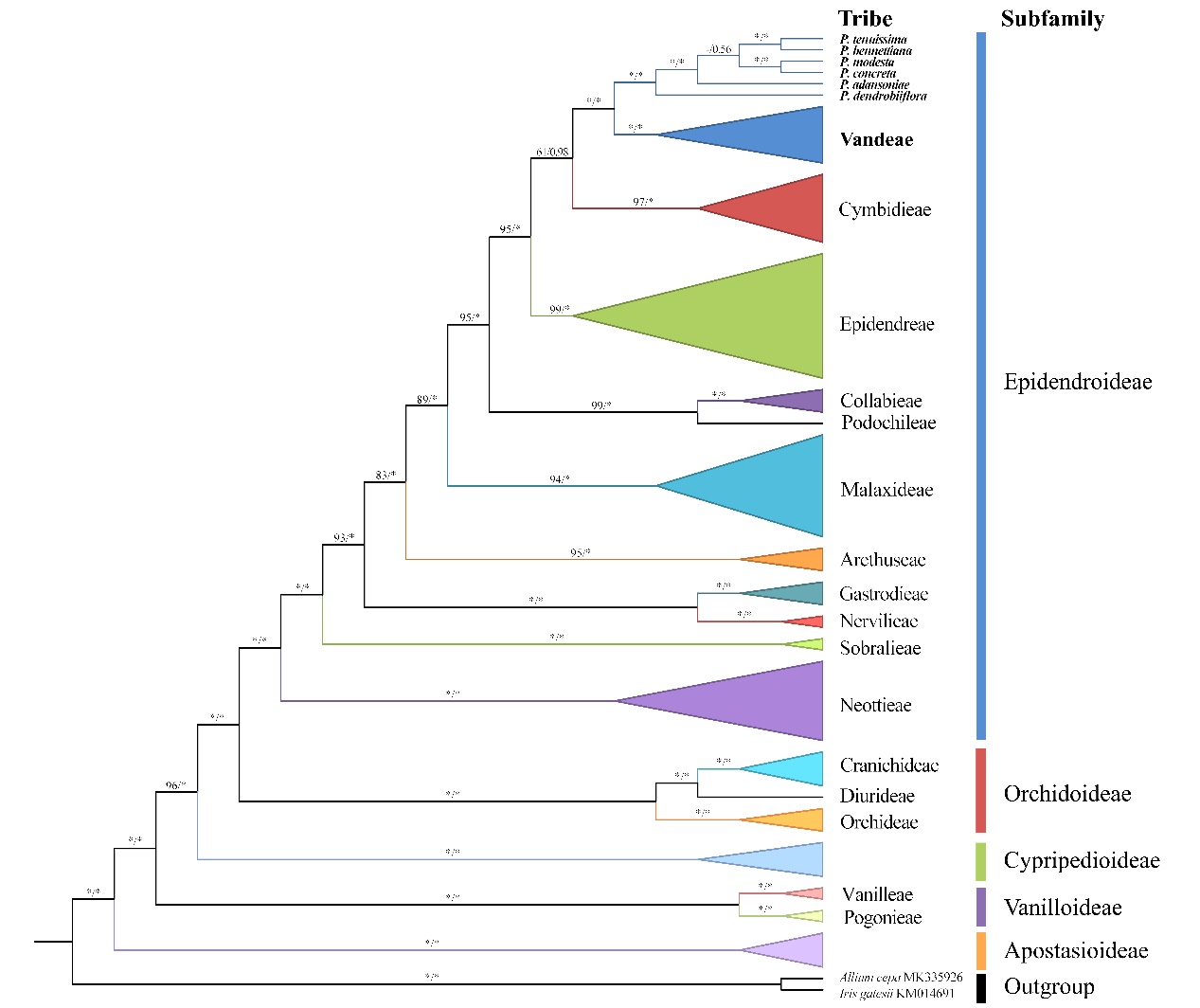


Figure S2. Phylogenetic tree constructed using ML and BI methods based on the 79 concatenate protein-coding sequences of whole cp genomes from 85 taxa. The numbers above the branches represent ML bootstrap values (BS)/BI posterior probabilities (PP). “*” indicates BS=100% or PP=1.00. “-” indicates BS<50% or PP<0.50. Different colored branches represent different subfamilies and tribes of Orchidaceae. The subfamily and tirbe circumscription of Orchidaceae is according to Chase et al. (2015).
