# Supplementary material for: Comparative and phylogenetic analyses of six Kenya Polystachya (Orchidaceae) species based on the complete chloroplast genome sequences
Source: BMC Plant Biol. 2022 Apr 6;22:177. doi: 10.1186/s12870-022-03529-5 (PMC8985347; doi:10.1186/s12870-022-03529-5)
Supplement: Supplementary file 1 — Additional file 1: Table S1. Taxonomic and GenBank accession information forsamples used for phylogenetic analyses (85). Table S2 and S3. Types of genes annotation and the intron-containing genes within the chloroplastgenomes of six Polystachya species. Table S4. Codonusage within the chloroplast genomes of six Polystachyaspecies. Table S5. Typesand amounts of SSRs within the chloroplast genomes of six Polystachya species. Table S6. Locationof repeat sequences within the chloroplast genomes of six Polystachya species. Table S7. Comparisonof site models for the 68 shared CDSs in the chloroplast genomes of six Polystachyaspecies and results of LRT. Table S8. Positiveselection sites based on BEB analysis in the M8 model detected in thechloroplast genomes of six Polystachya species. Table S9. Theoverall view of all gene alignment in the complete chloroplast genomes of six Polystachyaspecies. Table S10. Phylogenetictree constructed using ML and BI methods, based on the first and second codonpositions of 79 CDSs of whole cp genomes from 85 taxa. [file 12870_2022_3529_MOESM1_ESM.zip › Table S2 and S3.docx]

Table S2. Genes annotation for the chloroplast genome of the six *Polystachya* species

| Category for genes | Group of gene | Name of gene |
| --- | --- | --- |
| Genes for Genetic | Large subunit of ribosome  (LSU) | *rpl2**(×2), *rpl14*, *rpl16**, *rpl20*, *rpl22*, *rpl23*(×2), *rpl32*, *rpl33*, *rpl36* |
| System | Small subunit of ribosome  (SSU) | *rps2*, *rps3*, *rps4*, *rps7*(×2), *rps8*, *rps11*, *rps12***(×2), *rps14*, *rps15*, *rps16*, *rps18*, *rps19* |
|  | DNA dependent RNA  polymerase | *rpoA*, *rpoB*, *rpoC1**, *rpoC2* |
|  | ribosomal RNA (rRNA) | *rrn4.5*(×2), *rrn5*(×2), *rrn16*(×2), *rrn23*(×2) |
|  | transfer RNA (tRNA) | *trnA*^-^*^UGC^**(×2), *trnC*^-^*^GCA^*, *trnD*^-^*^GUC^*, *trnE*^-^*^UUC^*, *trnF*^-^*^GAA^*,  *trnfM*^-^*^CAU^*, *trnG*^-^*^GCC^**, *trnG*^-^*^UCC^*, *trnH*^-^*^GUG^*, *trnI*^-^*^CAU^*(×2), *trnI*^-^*^GAU^**(×2), *trnK^-UUU^**, *trnL*^-^*^CAA^*(×2), *trnL*^-^*^UAA^**, *trnL^-UAG^*, *trnM^-CAU^*, *trnN^-GUU^*(×2), *trnP^-UGG^*, *trnQ^-UUG^*, *trnR^-ACG^*(×2), *trnR^-UCU^*, *trnS^-GCU^*, *trnS^-GGA^*, *trnS^-UGA^*, *trnT^-GGU^,* *trnT^-UGU^*, *trnV^-GAC^*(×2), *trnV^-UAC^**, *trnW^-CCA^*, *trnY^-GUA^* |
|  | Translational  initiation factor | *infA* |
| Genes for | Photosystem I | *psaA*, *psaB*, *psaC*, *psaI*, *psaJ* |
| Photosynthetic | Photosystem II | *psbA*, *psbB*, *psbC*, *psbD*, *psbE*, *psbF*, *psbH*, *psbI*, *psbJ*,  *psbK*, *psbL*, *psbM*, *psbN*, *psbT*, *psbZ* |
| System | NADH dehydrogenase | ψ*ndhA*^1^*,* ψ*ndhB*(*×2*)^1^*,* ψ*ndhD*^1^*,* ψ*ndhE*^2^*,* ψ*ndhF*^3^*,* ψ*ndhG*^2^*,* ψ*ndhH*^4^*,* ψ*ndhI*^2^ |
|  | Cytochrome b / f complex | *petA*, *petB**, *petD**, *petG*, *petL*, *petN* |
|  | ATP synthase | *atpA*, *atpB*, *atpE*, *atpF**, *atpH*, *atpI* |
|  | Large subunit of Rubisco | *rbcL* |
| Genes for | Maturase | *matK* |
| Biosynthesis | ATP-dependent protease  proteolytic subunit | *clpP*** |
|  | Envelop membrane protein | *cemA* |
|  | Acetyl-CoA-carboxylase | *accD* |
|  | C-type cytchrome  synthesis gene | *ccsA* |
|  | Open Reading Frames of  unknown function | *ycf1*, *ycf2*(×2), *ycf3***, *ycf4* |

*: contains one intron; **: contains two introns; (×2): genes located in IRs; ψ: pseudogene; rps12: trans-splicing gene; ^1^: pseudogene in all six *Polystachya* species; ^2^: pseudogene in *P. adansoniae* only; ^3^: pseudogene in *P. dendrobiiflora*, *P. adansoniae*, *P.tenuissima*, *P. bennettiana*; ^4^: pseudogene in *P. dendrobiiflora*, *P.tenuissima*, *P. bennettiana*, *P. modesta*, *P. concreta*

Table S3. The genes with introns and the lengths of exons and intron

| Gene | Location | Exon Ⅰ (bp) | Intron Ⅰ (bp) | Exon Ⅱ (bp) | Intron Ⅱ (bp) | Exon Ⅲ (bp) |
| --- | --- | --- | --- | --- | --- | --- |
| *trnK^-UUU^* | LSC | 35 | 2762-2902 | 37 |  |  |
| *rps16* | LSC | 203-245 | 841-885 | 40 |  |  |
| *trnG^-GCC^* | LSC | 23 | 704-710 | 48 |  |  |
| *atpF* | LSC | 410 | 931-968 | 145 |  |  |
| *rpoC1* | LSC | 1617 | 753-769 | 435 |  |  |
| *ycf3* | LSC | 153 | 756-770 | 228 | 715-769 | 126 |
| *trnL^-UAA^* | LSC | 35 | 601-644 | 50 |  |  |
| *trnV^-UAC^* | LSC | 35 | 583-597 | 39 |  |  |
| *clpP* | LSC | 252-258 | 665-688 | 292 | 823-966 | 69 |
| *petB* | LSC | 6 | 696-726 | 642 |  |  |
| *petD* | LSC | 8 | 850-869 | 484 |  |  |
| *rpl16* | LSC | 399 | 1118-1243 | 9 |  |  |
| *rpl2* | IR | 431 | 663-677 | 385 |  |  |
| *trnI^-GAU^* | IR | 37 | 942-949 | 35 |  |  |
| *trnA^-UGC^* | IR | 38 | 796-808 | 35 |  |  |
| *rps12* | LSC | 114 | - | 26 | 539-547 | 232 |

red: represents two introns; blue: represents different intron lengths
