# Supplementary material for: Comparative and phylogenetic analyses of six Kenya Polystachya (Orchidaceae) species based on the complete chloroplast genome sequences
Source: BMC Plant Biol. 2022 Apr 6;22:177. doi: 10.1186/s12870-022-03529-5 (PMC8985347; doi:10.1186/s12870-022-03529-5)
Supplement: Supplementary file 1 — Additional file 1: Table S1. Taxonomic and GenBank accession information forsamples used for phylogenetic analyses (85). Table S2 and S3. Types of genes annotation and the intron-containing genes within the chloroplastgenomes of six Polystachya species. Table S4. Codonusage within the chloroplast genomes of six Polystachyaspecies. Table S5. Typesand amounts of SSRs within the chloroplast genomes of six Polystachya species. Table S6. Locationof repeat sequences within the chloroplast genomes of six Polystachya species. Table S7. Comparisonof site models for the 68 shared CDSs in the chloroplast genomes of six Polystachyaspecies and results of LRT. Table S8. Positiveselection sites based on BEB analysis in the M8 model detected in thechloroplast genomes of six Polystachya species. Table S9. Theoverall view of all gene alignment in the complete chloroplast genomes of six Polystachyaspecies. Table S10. Phylogenetictree constructed using ML and BI methods, based on the first and second codonpositions of 79 CDSs of whole cp genomes from 85 taxa. [file 12870_2022_3529_MOESM1_ESM.zip › Table S9.docx]

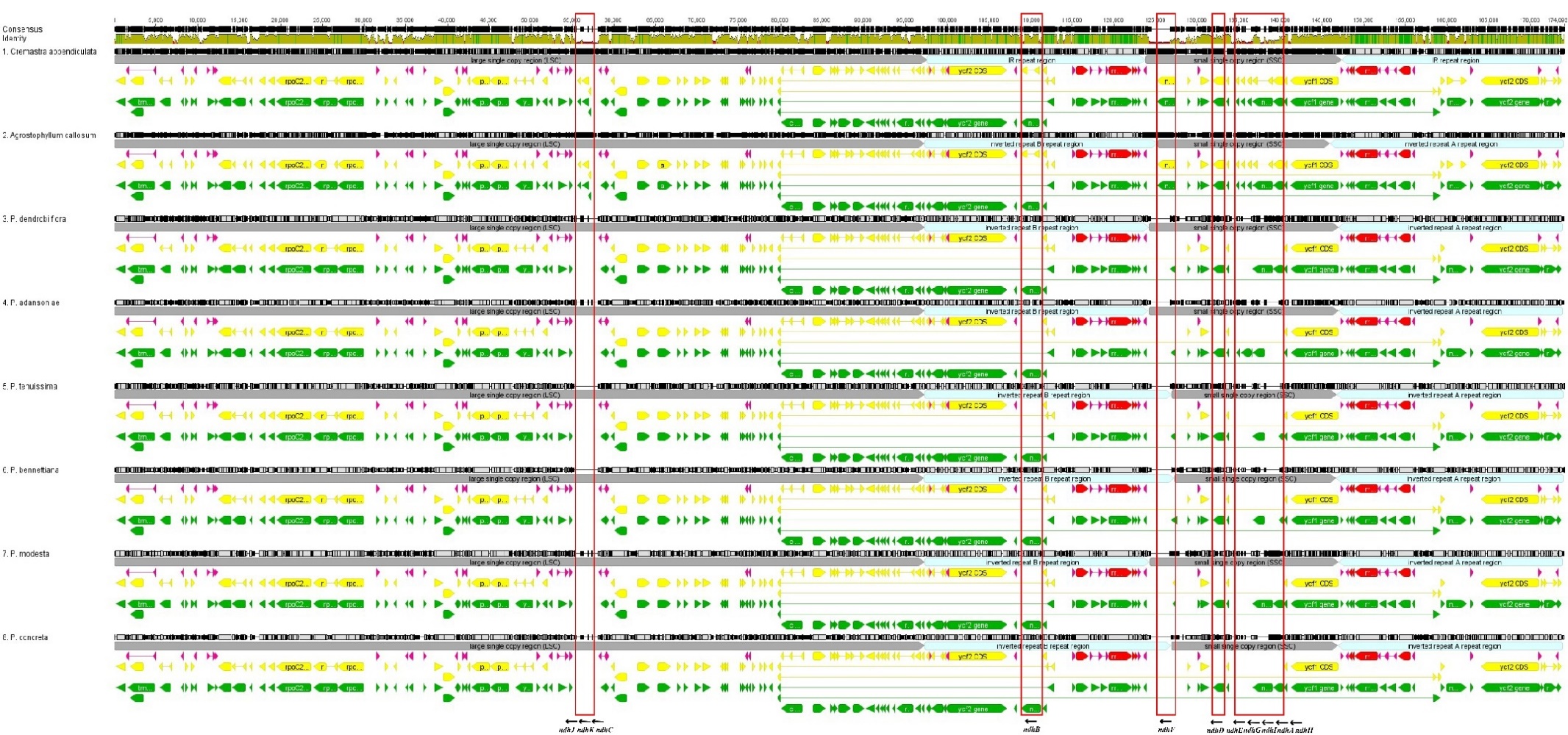


Figure S1. The overall view of all gene alignment in the complete chloroplast genomes of six *Polystachya* species, with containing all *ndh* genes and closely related *Cremastra appendiculata* and *Agrostophyllum callosum* as references. The consensus identity: the green color representing all sequences in columns have identical nucleotides, and the yellow color representing one or multiple sequences in columns have different nucleotides by mutations or short insertions/deletions, and the red color or line representing some sequences in columns have long insertions/deletions. The colored solid boxes denote all annotated genes from the chloroplast genome, and the red hollow boxes show the location of the eleven *ndh* genes.


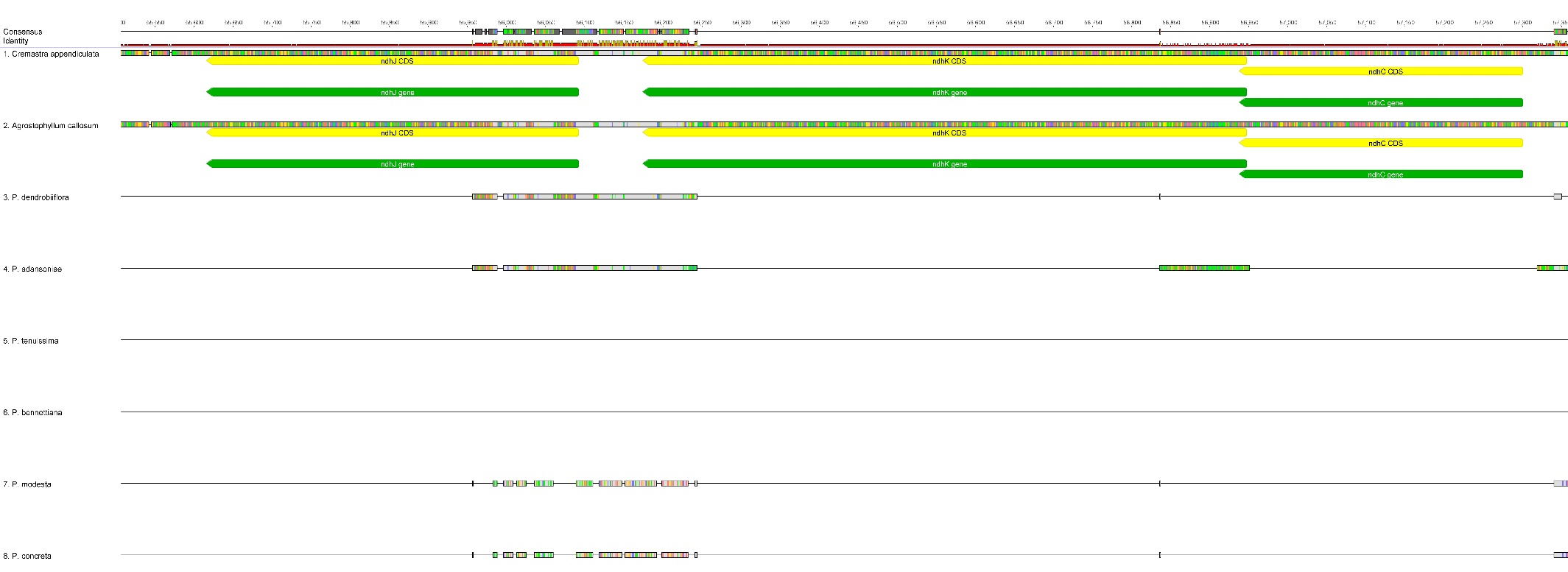


**A**


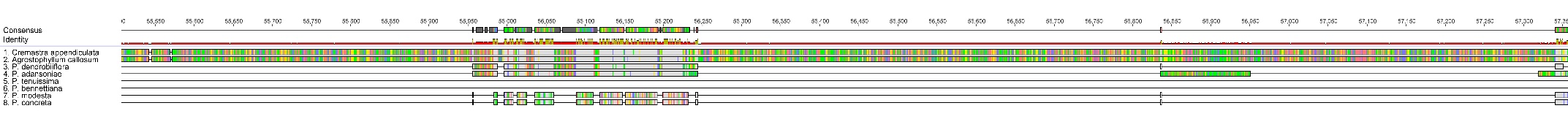


**B**

Figure S2. Screenshots of *ndhJ*, *ndhK* and *ndhC* genes regions alignment. (A). Screenshot of sequence alignment with gene annotation; (B). Screenshot of sequence alignment without gene annotation. The consensus identity: the green color means all sequence codes were consistent, the yellow color means some sequence codes were inconsistent (mutation or insertion), and the red color or line means some sequence codes were lost.


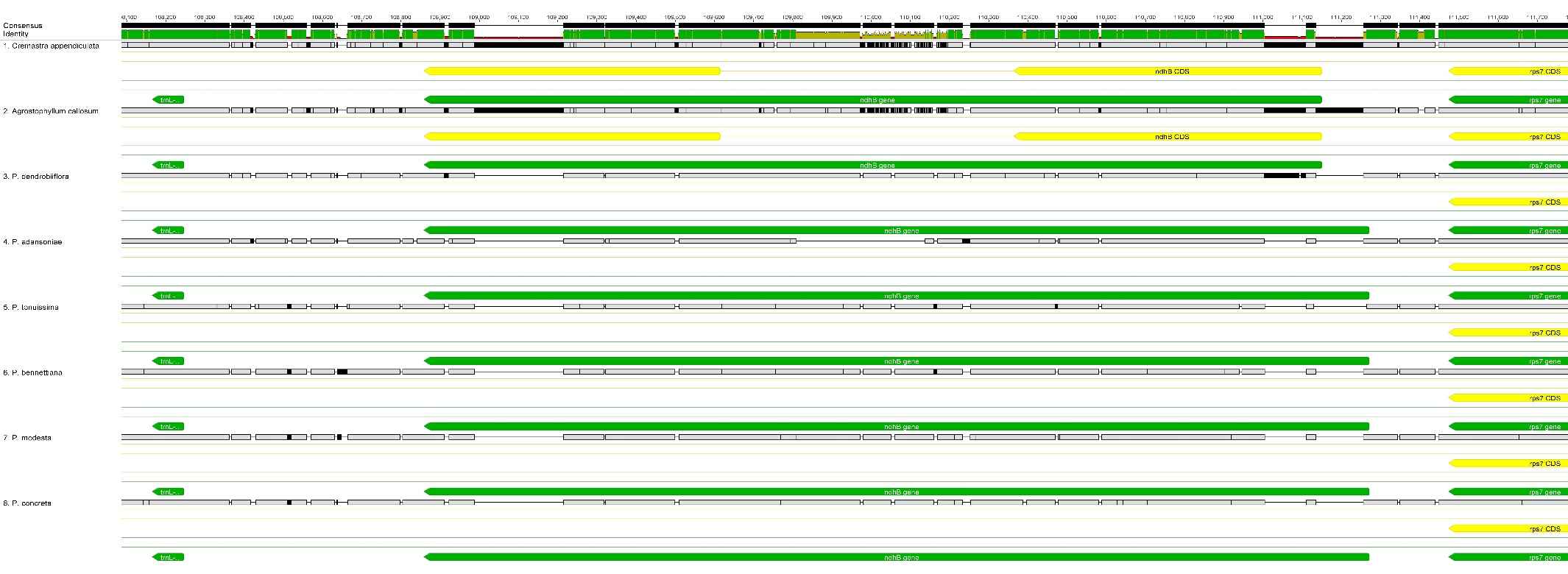


**A**


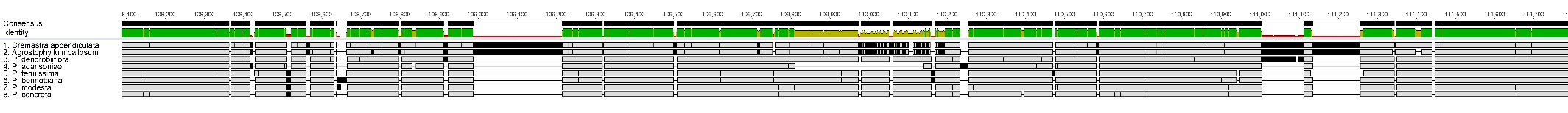


**B**

Figure S3. Screenshots of *ndhB* gene region alignment. (A). Screenshot of sequence alignment with gene annotation; (B). Screenshot of sequence alignment without gene annotation. The consensus identity: the green color means all sequence codes were consistent, the yellow color means some sequence codes were inconsistent (mutation or insertion), and the red color or line means some sequence codes were lost.


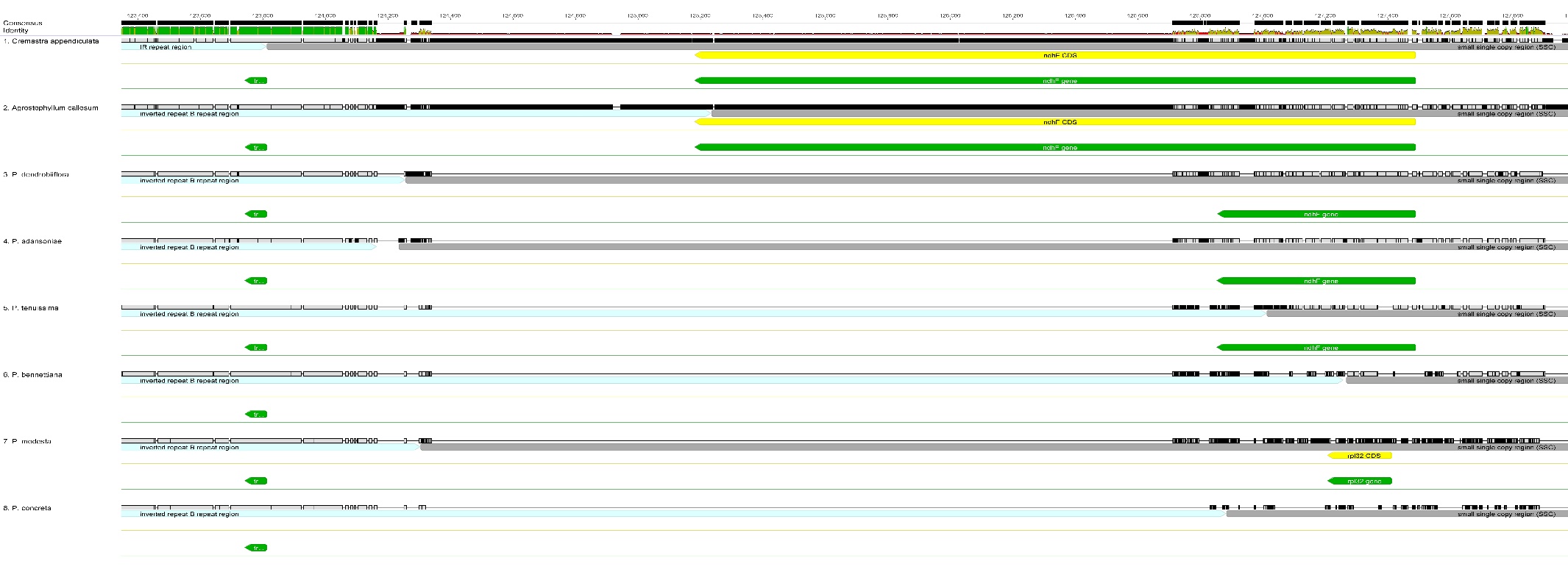


**A**


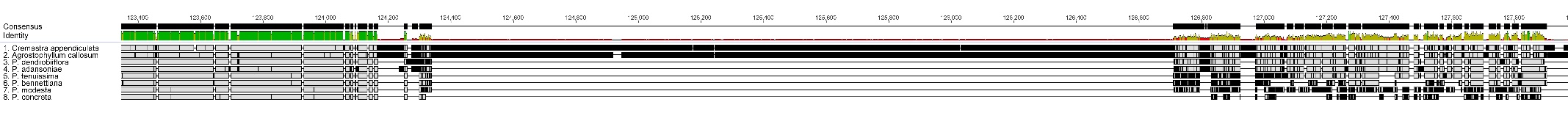


**B**

Figure S4. Screenshots of *ndhF* gene region alignment. (A). Screenshot of sequence alignment with gene annotation; (B). Screenshot of sequence alignment without gene annotation. The consensus identity: the green color means all sequence codes were consistent, the yellow color means some sequence codes were inconsistent (mutation or insertion), and the red color or line means some sequence codes were lost.


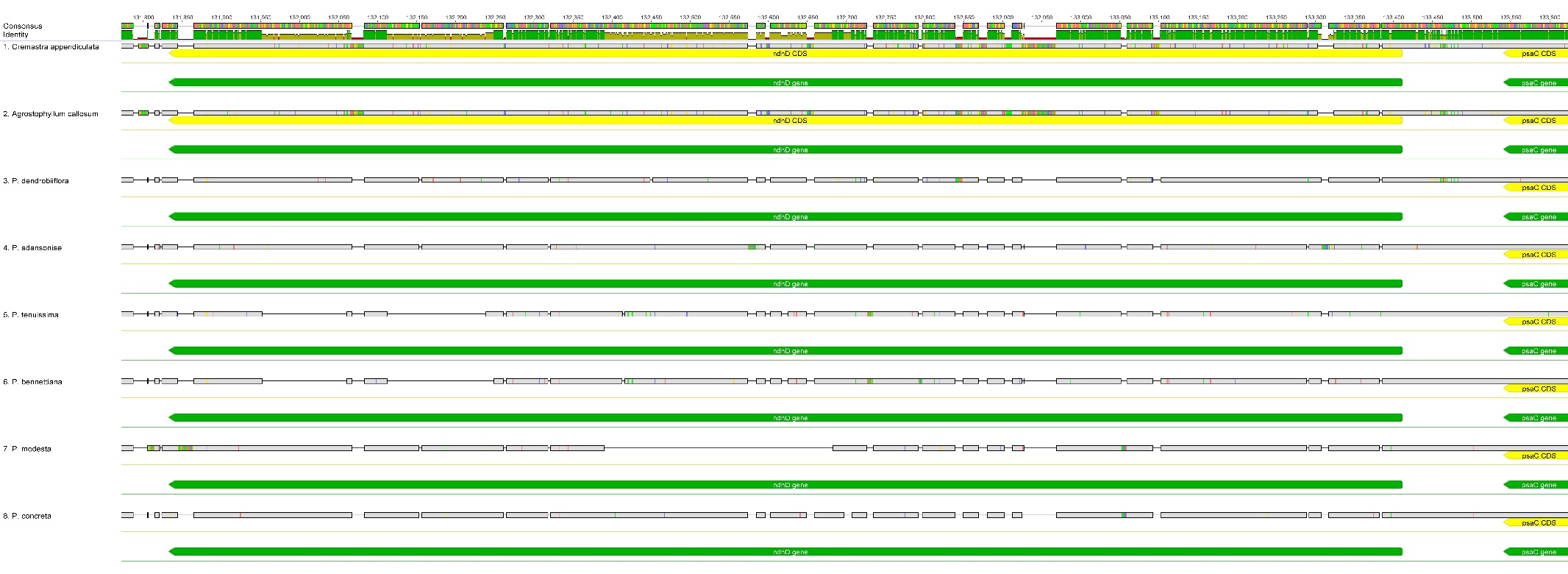


**A**


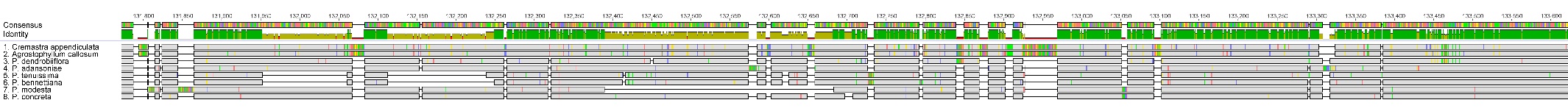


**B**

Figure S5. Screenshots of *ndhD* gene region alignment. (A). Screenshot of sequence alignment with gene annotation; (B). Screenshot of sequence alignment without gene annotation. The consensus identity: the green color means all sequence codes were consistent, the yellow color means some sequence codes were inconsistent (mutation or insertion), and the red color or line means some sequence codes were lost.


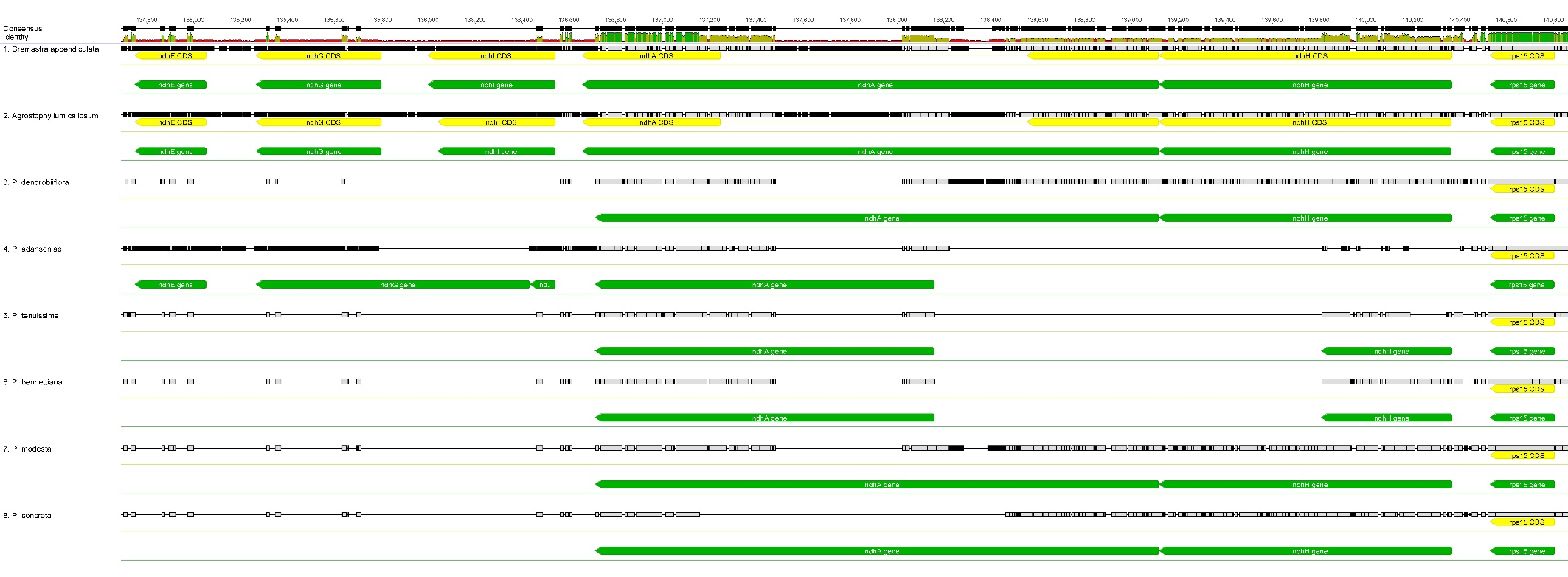


**A**


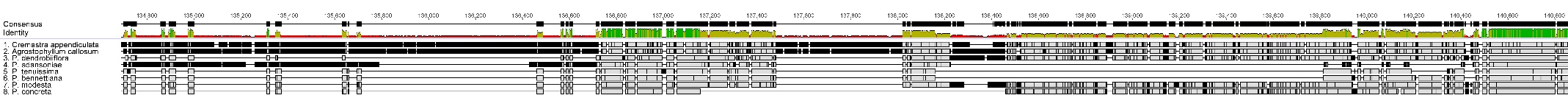


**B**

Figure S6. Screenshots of *ndhE*, *ndhG*, *ndhI*, *ndhA* and *ndhH* genes regions alignment. (A). Screenshot of sequence alignment with gene annotation; (B). Screenshot of sequence alignment without gene annotation. The consensus identity: the green color means all sequence codes were consistent, the yellow color means some sequence codes were inconsistent (mutation or insertion), and the red color or line means some sequence codes were lost.


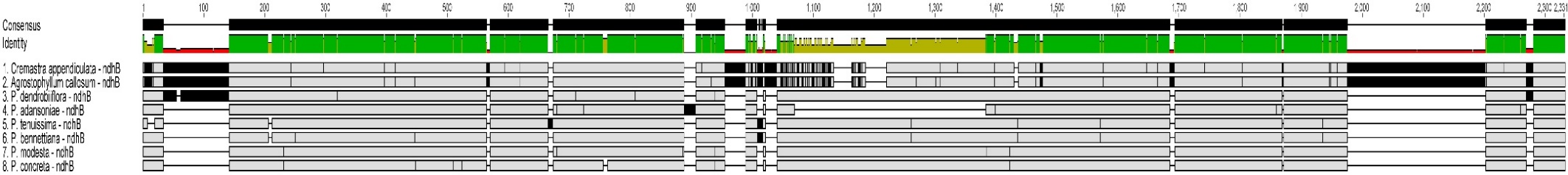


**A**


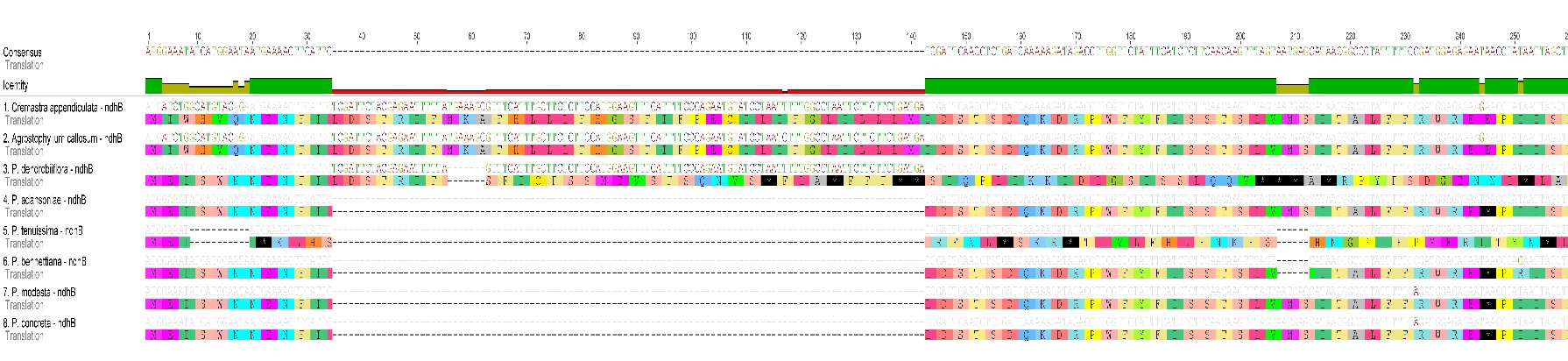


**B**

Figure S7. The overall view of *ndhB* gene region alignment in six *Polystachya* species, with containing normal *ndh* genes and closely related *Cremastra appendiculata* and *Agrostophyllum callosum* as references (A). Screenshot showing the starting position of *ndhB* gene alignment that stops codon generation or reads frame shift (B). The consensus identity: the green color representing all sequences in columns have identical nucleotides, and the yellow color representing one or multiple sequences in columns have different nucleotides by mutations or short insertions/deletions, and the red color or line representing some sequences in columns have long insertions/deletions.


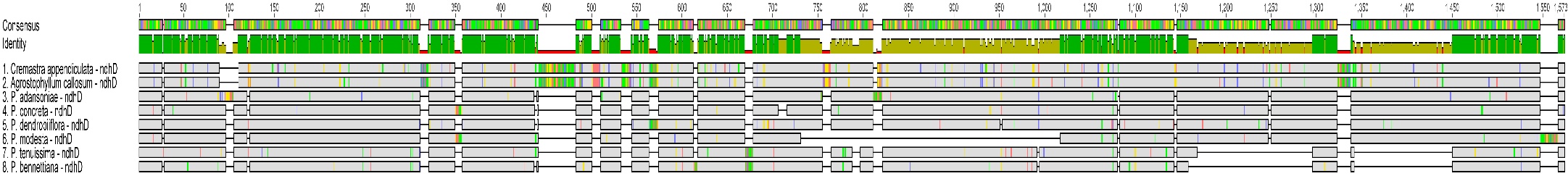


**A**


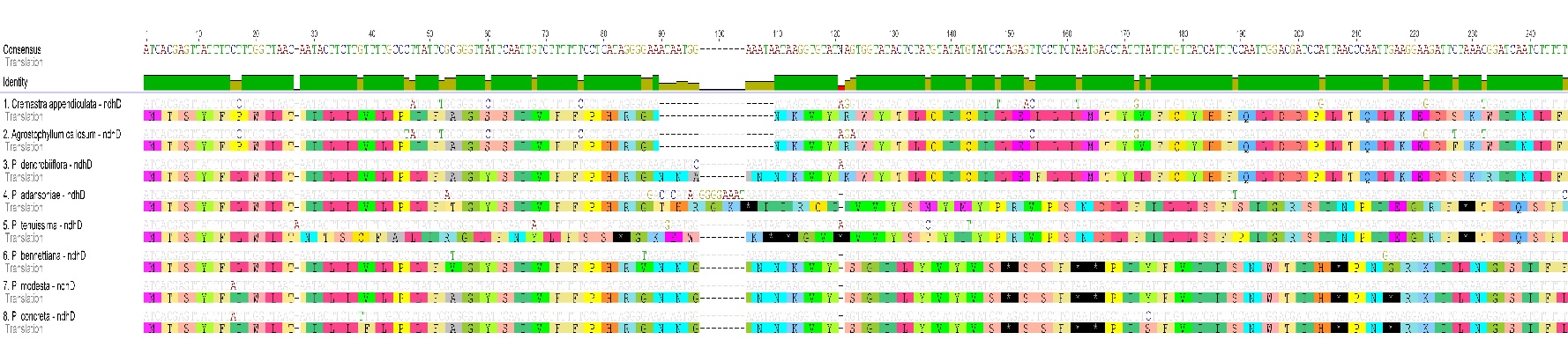


**B**

Figure S8. The overall view of *ndhB* gene region alignment in six *Polystachya* species, with containing normal *ndh* genes and closely related *Cremastra appendiculata* and *Agrostophyllum callosum* as references (A). Screenshot showing the starting position of *ndhB* gene alignment that stops codon generation or reads frame shift (B). The consensus identity: the green color representing all sequences in columns have identical nucleotides, and the yellow color representing one or multiple sequences in columns have different nucleotides by mutations or short insertions/deletions, and the red color or line representing some sequences in columns have long insertions/deletions.
